# I﻿nfrared thermal imaging to determine temperature parameters of dengue vector habitats across ecological regions of Nepal: a pilot feasibility study

**DOI:** 10.1038/s41598-026-61694-1

**Published:** 2026-07-27

**Authors:** Louise A. Kelly-Hope, Ajit Kumar Karna, Bijay Bajracharya, Satya Raj Paudel, Srijana KC, Pooja Shah, Matthew Baylis, Gokarna Dahal, Deena Shrestha

**Affiliations:** 1 Institute of Infection, Veterinary and Ecological Sciences, University of Liverpool, Liverpool, UK; 2 Center for Health and Disease Studies, Kathmandu, Nepal; 3 Epidemiology and Disease Control Division, Department of Health Services, Ministry of Health and Population, Kathmandu, Nepal

**Keywords:** Nepal, Mosquito vectors, Dengue, Arbovirus, *Aedes* spp., Larvae, Breeding sites, Temperature, *Culex* spp., Entomology, Thermal imaging, Thermometry, Ecology, Ecology, Environmental sciences, Zoology

## Abstract

Dengue is a major vector-borne disease in Nepal, driven by container-breeding *Aedes* mosquitoes that thrive in domestic, urban settings. Its risk is increasing from the lowland Terai to the Hill and Mountain regions due to urbanisation and global warming. Practical and innovative approaches are needed to better understand the impact on *Aedes* mosquito habitats that are the main targets for vector control. We conducted a pilot feasibility study to characterise *Aedes aegypti* and *Aedes albopictus* breeding sites, examine physicochemical parameters (total dissolved solids, pH, salinity, dissolved oxygen), and explore the utility of infrared thermal imaging as a novel tool for measuring temperature parameters of habitats across distinct ecological regions. We found that plastic containers, tin/metal items, and rubber tyres were common breeding sites. The dengue vectors *Ae. aegypti* and *Ae. albopictus* were identified in a variety of container types across most locations. Overall, there was reasonable agreement between the thermal camera and standard thermometer, although the camera recorded approximately 1 °C lower and showed divergence at higher temperatures. Most aquatic habitat temperatures were within an optimal mosquito breeding range (mid-20s to low 30s °C) and lower than non-breeding site temperatures. The breeding site water temperatures in the Terai region were ~ 4 °C higher than the Mid-Hill and Mountain regions. Aquatic habitat temperatures were also found to be significantly correlated with dissolved oxygen. Thermal imaging case studies provided key visual insights into varying temperatures in complex *Aedes* microclimates. Notably, inner rubber tyre temperatures remained relatively stable despite significant external temperature fluctuations and external tyre temperatures exceeding 50 °C. Thermal imaging shows promise for the field characterisation of mosquito breeding sites, but it is not interchangeable with standard thermometry without qualification, including calibration and uncertainty analysis.

**Supplementary Information:**

The online version contains supplementary material available at 10.1038/s41598-026-61694-1.

## Introduction

Nepal has experienced a surge in vector-borne diseases in recent years, most notably dengue, an important neglected tropical disease^1^. Dengue is transmitted to humans by infected *Aedes aegypti* (Linnaeus, 1762) and *Aedes albopictus* (Skuse, 1894) mosquitoes, which thrive in urban and peri-urban environments^2,3^. Dengue initially presented in Nepal in the early 2000s with sporadic cases and small outbreaks^1^. However, since 2021, when 540 cases were reported, dengue has dramatically increased by 77–100 times, reaching 54,784 cases in 2022, 52,790 cases in 2023, and 41,865 cases in 2024. This surge in disease has placed considerable strain on the public health system, and led to an urgent need for better preparedness and response^1,4–6^. These vectors belong to the genus *Aedes* and also transmit chikungunya virus, an important emerging arbovirus in Nepal, but with limited data^7^.

Nepal is ecologically diverse. The lowland Terai region, mostly below 200 m above sea level, has the highest risk of dengue and other vector-borne diseases. This is due to the tropical and subtropical climate including higher temperatures, rainfall and humidity, which can facilitate the survival, breeding, and feeding habits of vectors^8–10^. However, environmental and climate change are increasing the risk of dengue for people living at high altitudes in the Hill and Mountain regions, as the *Aedes* vectors expand their geographical range^11–13^. For example, in 2022, the highest number of dengue cases were recorded in Kathmandu, the capital city at 1400 m above sea level^1,4^. In 2025, thousands of cases were reported across the country, with cases reported in all districts except Dolpa District in the Mountain region^1^.

Preventative measures for dengue are based on vector control with the aim to reduce the aquatic habitats of *Aedes* larvae by targeting water containers in the domestic and surrounding environment^2,3^. Understanding the local ecosystem and implementing community-based approaches has been shown to be effective for reducing *Aedes* populations in Asian endemic countries^14,15^. In Nepal, recent entomological studies highlight that *Ae. aegypti* and *Ae. albopictus* are widespread, breeding in a range of containers (e.g., rubber tyres, plastic bottles), and that there is evidence of infection with dengue viru*s*^9,10,16–19^. Additional entomological studies are required across the various ecological zones taking into account the environmental and climatic changes^20,21^.

Nepal is one of the most vulnerable countries in the world to climate change. Nepal’s National Adaptation Plan highlights several major vulnerabilities, including rising temperatures, erratic rainfall, increased climate hazards such as floods and fragile mountain ecosystems facing water scarcity in hills and mountains^22^. The country’s climate change vulnerability is compounded by its rugged terrain and low levels of development, leaving millions of people living in vector-thriving environments with limited or inadequate vector control activities^23–25^.

Global warming is expected to increase vector-borne diseases^26,27^. However, there is a general lack of research on how the warming temperatures across the different ecological regions may affect *Aedes* breeding sites. Temperature can affect all stages of the life cycle of the mosquito: egg, larva, pupa, and adult, and therefore directly influence the transmission dynamics and risk of infection^3^. Research has mainly focussed on air temperature under controlled experimental conditions, or ambient temperature (i.e., outdoor environment), with optimal ranges for larval development considered to be mid-20s to low 30s °C. The effects of higher temperatures are multifaceted and can lead to varying outcomes. For example, increased temperatures may accelerate larval development, whereas very high temperatures i.e., > 35 °C, may be too hot for larvae and cause stress, suppress development, decrease fecundity, and increase mortality^28–31^.

The relationship between air or ambient temperature and mosquito larval habitats is not well understood. A study in India found that temperatures of mosquito aquatic habitats were lower than air temperatures, and varied by container type, with plastic containers recording the highest (17.3–35.6 °C) and ceramic pots (14.3–28.3 °C) the lowest temperatures^32^. Similar trends in *Aedes* container temperatures were observed in laboratory studies, with optimal larval development temperatures between 23.6 and 32.2 °C^33^. Notably, temperatures > 32.2 °C delayed *Ae. aegypti* development and > 35.0 °C caused mortality ^33^. Additional studies in Asia have observed similar aquatic habitat temperature ranges; in Sri Lanka 25.3–39.8 °C^34^, in Malaysia 28.5–29.3 °C^35^, and in Indonesia 27–32 °C^36^. These thermal patterns align with established literature, although larval development is also influenced by or interacts with other factors such as humidity, resource availability, larval density, competition, habitat conditions, and geographic origin^30,37–39^.

Further research is needed to better understand the temperature parameters of *Aedes* aquatic habitats and their immediate surrounding areas to aid public health planning globally^40^. Temperatures are typically measured directly using standard thermometers, or digital probes. However, the use of infrared thermometers or thermal imaging may provide a novel way to assess and visualise water and surrounding surface temperature parameters. Infrared thermography has been used in a wide range of biological and medical studies^41–44^, including *Aedes* mosquitoes and their host seeking behaviour^45^, and as point-of-care tools for the neglected tropical diseases lymphatic filariasis and snakebite^46,47^. A range of digital infrared thermal cameras are available that are compact, robust, and may be practical for fieldwork.

Here, we aimed to characterise *Ae. aegypti* and *Ae. albopictus* breeding sites, examine physicochemical parameters (total dissolved solids, pH, salinity, dissolved oxygen), and explore the utility of an infrared thermal camera as a tool to measuring temperature parameters of habitats across distinct ecological regions of Nepal. The study was broadly categorised into two main parts with six main objectives listed below. The first part focused on environmental parameters, assessment of temperature measurement tools, and breeding site characteristics and temperatures. The second part focussed on mosquito species identification, *Aedes*-specific breeding site water temperatures, and *Aedes*-specific infrared thermal imaging cases studies.

Part 1 specific objectives:

1. determine ambient conditions and breeding site water temperature and physicochemical parameters
2. assess breeding site water temperature agreement between the infrared thermal camera and standard thermometer and associations with physicochemical parameters
3. determine breeding sites characteristics and their infrared thermal water temperature parameters

Part 2 specific objectives.

4. identify the range of mosquito species and their breeding site characteristics
5. determine *Ae. aegypti* and *Ae. albopictus* breeding site infrared thermal water temperature parameters
6. present case studies as visual examples demonstrating the potential of the method to assess infrared thermal temperatures across different vector microclimates

## Methods

### Study design and sampling methodology

A cross-sectional pilot feasibility study was conducted during the Monsoon (rainy) season coinciding with the peak dengue transmission period, and to assess potential mosquito breeding habitats in four districts across distinct ecological regions. In each district, one location (e.g. ward, suburb) was selected based on evidence of dengue risk in national reports^1^. The location, district, settlement type (i.e. urban or rural), ecological zone (i.e., Terai, Mid-Hill/Hill, Lower Mountain), and broad ecological descriptions of climate and vegetation are summarised in Table 1^48,49^.

**Table 1:** Summary of the four study locations.

Location, district	Urban/Rural	Elevation (metres)	Ecological zone	Ecological description
Kshetrapur, Chitwan	Urban	< 200	Terai	Tropical bioclimatic zone, lowland plains, savanna and grasslands, average annual rainfall 1100-3000 mm, average annual temperature 20–25 °C
Baneshwor, Kathmandu	Urban	1300–1400	Mid-hill	Subtropical bioclimatic zone, hills, broadleaf mixed forests, average annual rainfall 275-2300 mm, average annual temperature 10–20 °C
Gundu, Bhaktapur	Rural	1300–1400	Mid-hill	Subtropical bioclimatic zone, hills, broadleaf mixed forests, average annual rainfall 275-2300 mm, average annual temperature 10–20 °C
Devichaur, Lalitpur	Rural	> 1600	Mountain (Lower)	Subtropical / montane bioclimatic zone, hill to mountain transition area, broadleaf /temperate forests, average annual rainfall 275-2300 mm, average annual temperature 10–20 °C

Prior to the start of the survey, the research team communicated and coordinated with the local representatives from the ward (e.g., community leader) to inform the community representative of the study’s aim and objectives.

At each of the four study locations, between 15 and 25 households were randomly selected from a central location. If a multiple storeyed building was selected, only the ground floor was considered for selection. Informed verbal consent was obtained from the household head before the inspection of the mosquito habitats.

The main survey was conducted between 7:00 and 12:00 local time each day and is hereafter referred to as the morning period. Within and around each household, potential mosquito breeding sites were systematically identified and assessed. To compare breeding site temperatures at different times of the day, a small subset of selected households was revisited and sampled between 12:00 and 14:00 local time, hereafter referred to as the midday period.

### Field data and assessments of breeding sites and the local environment

During the field work, data were collected on four main aspects of breeding sites and the local environment. The data, key characteristics and methods are described below.

**Fig. 1:**
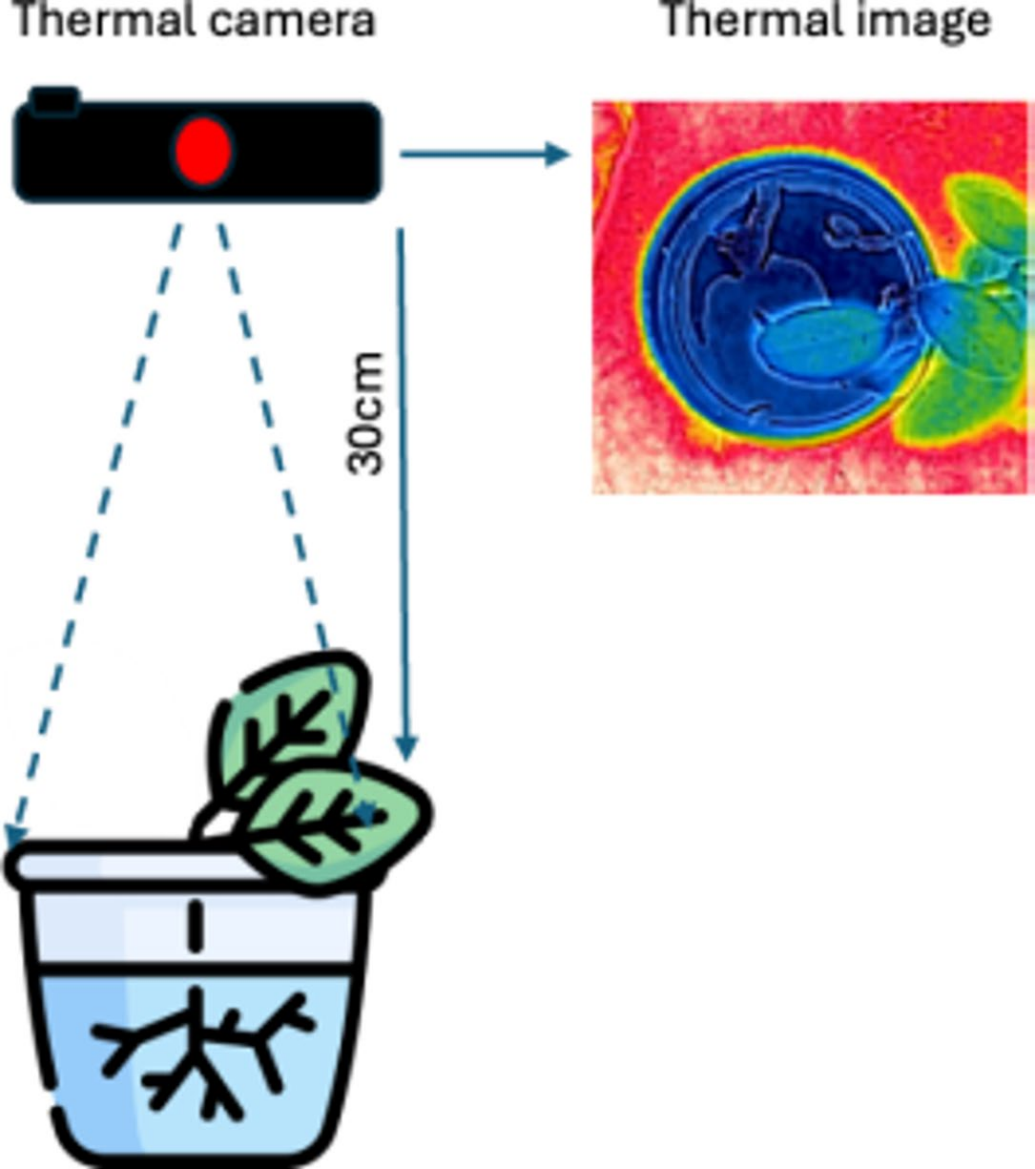
Graphic showing the thermal camera held above the potential mosquito breeding site Plant pot Image: Adapted from Flaticon.com.

1. Ambient temperature and humidity measurements at each household were measured during the entomological survey using a portable indoor hygrometer (temperature range − 50 to 70 °C and relative humidity range 10 to 99%).
2. Breeding site physicochemical parameters were assessed using the following measurements.
  i) Water temperature using a standard digital thermometer, dipped in the water just below the surface approximately 2 cm and not more than 5 cm deep.
  ii) Water total dissolved solids (TDS) of all organic/inorganic material, pH to gauge acidity/alkalinity, salinity to measure salt content, and dissolved oxygen (DO) using multiparameter water quality meters (model EZ-9909SP and Lutron WA-2015).

3. Breeding site characteristics were defined based on the following attributes^50^:
  i) Type: artificial (human-made) or natural (plant or soil).
  ii) Material: recorded and grouped into plastic, metal/tin, rubber, concrete, other.
  iii) Colour: recorded and grouped into dark coloured (black, blue, green) or light coloured (white, transparent, grey, silver).
  iv) Size: small (< 10 L.e.g., plant pot, cans), medium (10–100 L. e.g., bucket, tyres, clay pots), or large (> 100 L. e.g., cement tanks, wells).
  v) Debris: level of vegetation or organic matter present on the surface of the water estimated at ≤ 50% or > 50%.
  vi) Sunlight: exposure estimated at ≤ 50% or > 50%.

4. Breeding site infrared thermal images were captured using a FLIR C5 infrared thermal camera (Teledyne FLIR LLC, Oregon, USA), which can measure temperatures between 0–100 °C to an accuracy of ± 3 °C. The emissivity, which quantifies the effectiveness of a surface radiating energy on a scale from 0 (low) to 1 (high), was set at 0.90 in the thermal camera to account for the mix of surfaces (e.g., water, plastic, rubber, concrete, glass, earth) and emissivity levels that range from approximately 0.85 to 0.96^51,52^. The Multi-Spectral Dynamic Imaging (MSX®) enhancement setting, a patented FLIR ® imaging technology that combines thermal and visible light, was used to optimise the images. We obtained and analysed data from measurements collected during and after the survey; methods are briefly described here.
  i) During the survey: Temperature of the breeding site water was recorded from an image captured by the thermal camera held approximately 30 cm (12 inches), directly above the site (see Fig. 1).
  ii) After the survey: Temperatures of the breeding site water, material, and immediate surrounding environment (1–5 m distance) were assessed from captured images and analysed in FLIR Thermal Studio Suite Teledyne (FLIR LLC, Oregon, USA) to assess the range of temperatures for a set of case studies.

### Specific data and image analysis for each objective

Field data were collated in Microsoft Excel (Version 16.107, Microsoft Corporation) and imported into IBM SPSS Statistics (Version 31.0.0.0) for statistical analyses and data graphics. Statistical significance was set at p < 0.05. For all objectives, data were stratified by each location to highlight differences between ecological regions.

Objective 1: Ambient conditions and breeding site water parameters.

- Ambient temperature and humidity measured at each surveyed household, and water temperature (thermometer and thermal camera) and physicochemical parameters of mosquito breeding sites, were summarised. Mean measurements were compared statistically using one-way ANOVA. The Tukey honestly significant difference (HSD) post hoc test was conducted to determine which specific groups differed from each other.
- Water temperatures of potential breeding sites (overall and for *Aedes* vectors) collected using the thermal camera were plotted using histograms with a normal distribution trend line applied across four study locations.

Objective 2: Breeding site water temperature agreement between the infrared thermal camera and standard thermometer and associations with physicochemical parameters.

- Water temperatures measured using a thermal camera were correlated with those obtained using a standard thermometer and the physicochemical parameters using Pearson’s correlation coefficient (r). The strength and direction of the relationship were assessed and visually presented using scatter plots.
- Agreement between temperatures obtained using the thermal camera and thermometer was assessed using Bland–Altman analysis. First, systematic bias was calculated as the mean difference to determine if one tool provided higher or lower values than the other; significant differences from zero were determined using a one-sample t-test. Second, the 95% limits of agreement (LoA) were defined as the mean difference ± 1.96 standard deviations (SD). Third, proportional bias was assessed using linear regression of the differences and average temperatures to determine if the differences increased or decreased in proportion to the temperature levels. Finally, outliers and heteroscedasticity (random scatter) were visually assessed to identify any unusual data points or patterns. Data were visualised using Bland–Altman plots.

Objective 3. Breeding sites characteristics and infrared thermal water temperature parameters.

- Key characteristics of potential mosquito breeding sites as described above (i.e., type, material, colour, size, debris, sunlight) were summarised by each study location. Frequency differences across the four locations were assessed using the Fisher’s exact test for 2 × 4 tables and the Fisher-Freeman-Halton test for 3 × 4 and 4 × 4 tables to account for expected small cell counts in this pilot study.
- Mean breeding water temperatures obtained using the thermal camera were summarised by each study location. Mean temperatures were compared statistically using one-way ANOVA with Tukey post hoc tests conducted to determine which specific groups differed from each other.
- A linear mixed effects model was used to assess the association between breeding site characteristics and water temperature. Water temperature was specified as the outcome variable. Breeding site location and characteristics, including material, colour, size, debris, and sunlight, were specified as fixed effects, along with their interaction terms. Household was included as a random effect to account for household clustering. Reference categories were Chitwan for location, rubber for material, light for colour, small for size, > 50% for debris, and > 50% for sunlight. Results are presented as regression coefficients, confidence intervals (95%) and p-values in the main text and supplementary file.

Objective 4: Mosquito species and their breeding site characteristics.

- The presence or absence of mosquito larvae and/or pupae in each potential breeding site was determined by collecting water with a ladle and pouring it through a fine mesh sieve into a tray to capture larvae or pupae. These were morphologically identified by a trained entomologist and classed as either larvae or pupae using a magnifying glass at the site of collection. Larvae and/or pupae were collected in clean falcon tubes and transported to an insectary in Kathmandu where they were reared to adults. The adult mosquitoes were morphologically identified to species level following standard taxonomic keys^53,54^.
- Mosquito species data were summarised across the study locations. The presence and absence of *Ae. aegypti* and *Ae. albopictus* vectors in relation to breeding site characteristics (i.e., type, material, colour, size, debris, sunlight) were assessed using the Fisher’s exact test for 2 × 2 tables and the Fisher-Freeman-Halton test for 2 × 3 tables.

Objective 5: *Ae. aegypti* and *Ae. albopictus* breeding site infrared thermal water temperature parameters.

- Water temperatures obtained using the infrared thermal camera of *Aedes* vector breeding sites were summarised by each location to highlight differences between ecological regions. Mean temperatures of each location were compared statistically using one-way ANOVA with Tukey post hoc tests conducted to determine which specific location differed from the other.
- An exploratory analysis of *Aedes* vector breeding site water temperatures at different times of day was conducted. Data were summarised for selected households in each location to highlight the range of temperature changes between the morning and midday periods. Selected breeding sites were identified to use for case studies (described below).

Objective 6. Case studies of infrared thermal temperatures across different microclimates.

- The thermal images of different types of mosquito breeding sites were assessed to identify visual examples for the case studies that included different locations, types of breeding site materials, and larvae/ pupae presence. Images included both thermal and standard photographs, with the temperatures of key points or measured areas highlighted and summarised. The utility of the FLIR Thermal Studio Suite Teledyne (FLIR LLC, Oregon, USA), and how temperature data may be summarised, was highlighted by presenting a mix of points or spots (depicted as SP) and polygons (depicted as Bx or El).

## Results

The field survey was conducted in July 2023 during the Monsoon (rainy) season. In total, 83 households were included in the assessment across the four locations (Chitwan = 21, Kathmandu = 23, Bhaktapur = 20, Lalitpur = 19), and a total of 171 potential breeding sites were identified and assessed (Chitwan = 36, Kathmandu = 50, Bhaktapur = 38, Lalitpur = 47).

### Objective 1

#### Ambient conditions and mosquito breeding site water parameters

Table 2 presents a summary of household level ambient temperature and humidity measurements, and breeding site water temperatures and physicochemical parameters across the four study locations.

**Table 2:** Summary of ambient temperature and humidity, and overall mosquito breeding site water parameters across the four study locations.

Ecological zone and study location	Terai Chitwan (urban) ≤ 200 m		Hills Kathmandu (urban) 1300-1400 m		Hills Bhaktapur (rural) 1300-1400 m		Mountain Lalitpur (rural) > 1600 m		Overall		p-value between locations (row)
Measure	Mean (SE)	No. of sites	Mean (SE)	No. of sites	Mean (SE)	No. of sites	Mean (SE)	No. of sites	Mean (SE)	No. of sites	
Household (HH) level											
		21 HH		23 HH		20 HH		19 HH		83 HH	
Ambient temperature	31.5^a^ (0.4)		29.6 (0.3)		29.8 (0.4)		30.4 (0.3)		30.3 (0.2)		p = 0.002*
Ambient humidity	80.7^b^ (1.5)		63.1 (1.2)		71.2^c^ (1.3)		63.1 (1.2)		68.6 (0.8)		p < 0.001**
Breeding site (BS) level											
		36 BS		50 BS		38 BS		47 BS		171 BS	
Thermometer water temperature	28.0^b^ (0.3)		24.4 (0.2)		24.7 (0.4)		24.3 (0.4)		25.2 (0.2)		p < 0.001**
Thermal camera water temperature	27.2^b^ (0.5)		23.2 (0.4)		23.5 (0.5)		23.4 (0.5)		24.2 (0.3)		p < 0.001**
Total dissolved solids (TDS)	199.0 (49.1)		269.5 (53.7)		514.5 (191.9)		362.9 (147.8)		334.8 (61.9)		p = 0.354
pH	7.5 (0.1)		7.9 (0.9)		9.8 (1.8)		7.9 (0.1)		8.2 (0.4)		p = 0.256
Salinity	0.016 (0.004)		0.035 (0.009)		0.045 (0.02)		0.033 (0.02)		0.033 (0.007)		p = 0.610
Dissolved oxygen (DO)	3.54^d^ (0.1)		3.02 (0.1)		3.80^e^ (0.2)		2.86 (0.1)		3.26 (0.8)		p < 0.001**

*Significant at the 0.05 level.

**Significant at the 0.01 level.

Summary of Tukey (HSD) tests.

^a^Chitwan measurements significantly higher than Kathmandu (p = 0.002) and Bhaktapur (p = 0.01)

^b^Chitwan measurements significantly higher than Kathmandu, Bhaktapur and Lalitpur (p < 0.001)

^c^Bhaktapur measurements significantly higher than Kathmandu and Lalitpur (p < 0.001)

^d^Chitwan measurements significantly higher than Lalitpur (p < 0.001)

^e^Bhaktapur measurements significantly higher than Kathmandu (p < 0.001) and Lalitpur (p < 0.001)

At household level, a one-way ANOVA showed significant differences in ambient temperature (*p* = 0.002) and humidity measurements (*p* < 0.001) across the study locations. Tukey HSD post hoc comparisons of ambient temperatures found that Chitwan, Terai region (31.5 °C) had significantly higher temperatures than Kathmandu (*p* = 0.002) and Bhaktapur (*p* = 0.010) in the Mid-Hill region (temperature range 29.6–29 °C). Post hoc comparisons of ambient humidity found that Chitwan (80.7) had significantly higher humidity than Kathmandu, Bhaktapur and Lalitpur (humidity range 63.1–71.2; *p* < 0.001), and that Bhaktapur had significantly higher humidity than Kathmandu and Lalitpur (*p* < 0.05). See supplementary file 1 for more detailed post hoc results.

**Fig. 2:**
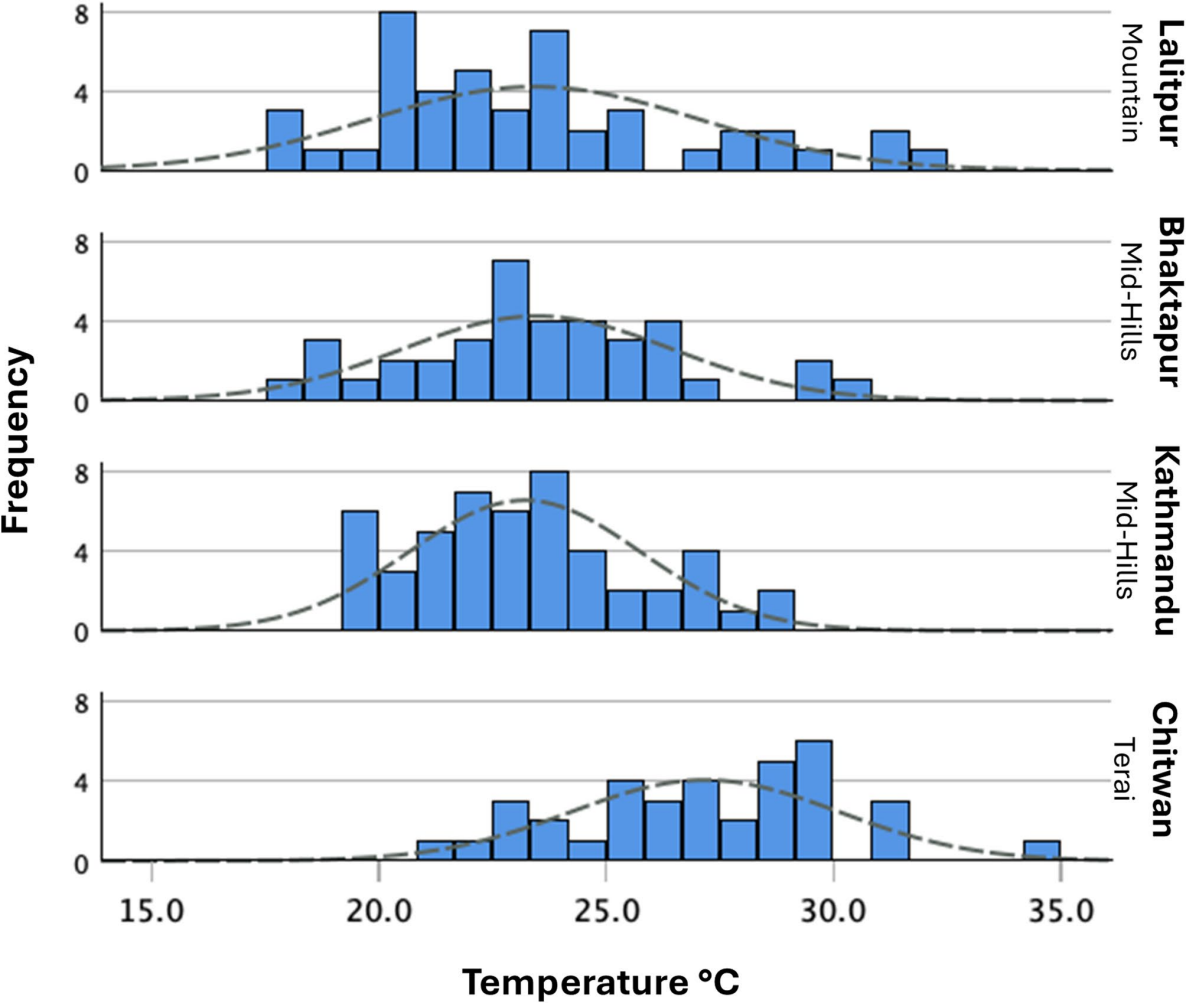
Range of thermal camera water temperatures of all potential mosquito breeding sites across the four study locations.

At breeding site level, a one-way ANOVA showed significant differences in breeding site water temperatures measured using a standard thermometer (*p* < 0.001) and using the thermal camera (*p* < 0.001) across study locations. Tukey HSD post hoc comparisons of water temperature found that Chitwan (thermometer 28.0 °C; thermal camera 27.2 °C) had significantly higher temperatures than Kathmandu, Bhaktapur and Lalitpur (thermometer range 24.3–25.2 °C; *p* < 0.001; thermal camera range 23.2–24.2 °C; *p* < 0.001). Thermal camera water temperature ranges of all potential breeding sites are shown in Fig. 2 and highlight the higher minimum and maximum temperature range in Chitwan, Terai region compared with the Mid-Hill and Mountain regions.

Physicochemical parameters of breeding site water varied across the four study locations. No significant differences were observed in total dissolved solids, pH, or salinity across locations.

A one-way ANOVA showed significant differences in dissolved oxygen parameters of breeding site water (*p* < 0.001) across the study locations. Tukey HSD post hoc comparisons found that Chitwan (3.54) had significantly higher levels of dissolved oxygen than Lalitpur (2.86; *p* < 0.001), and that Bhaktapur (3.80) had significantly higher levels than Kathmandu (3.02; *p* < 0.001) and Lalitpur (2.86; *p* < 0.001).

### Objective 2

#### Breeding site water temperature agreement between the infrared thermal camera and standard thermometer and associations with physicochemical parameters

**Fig. 3:**
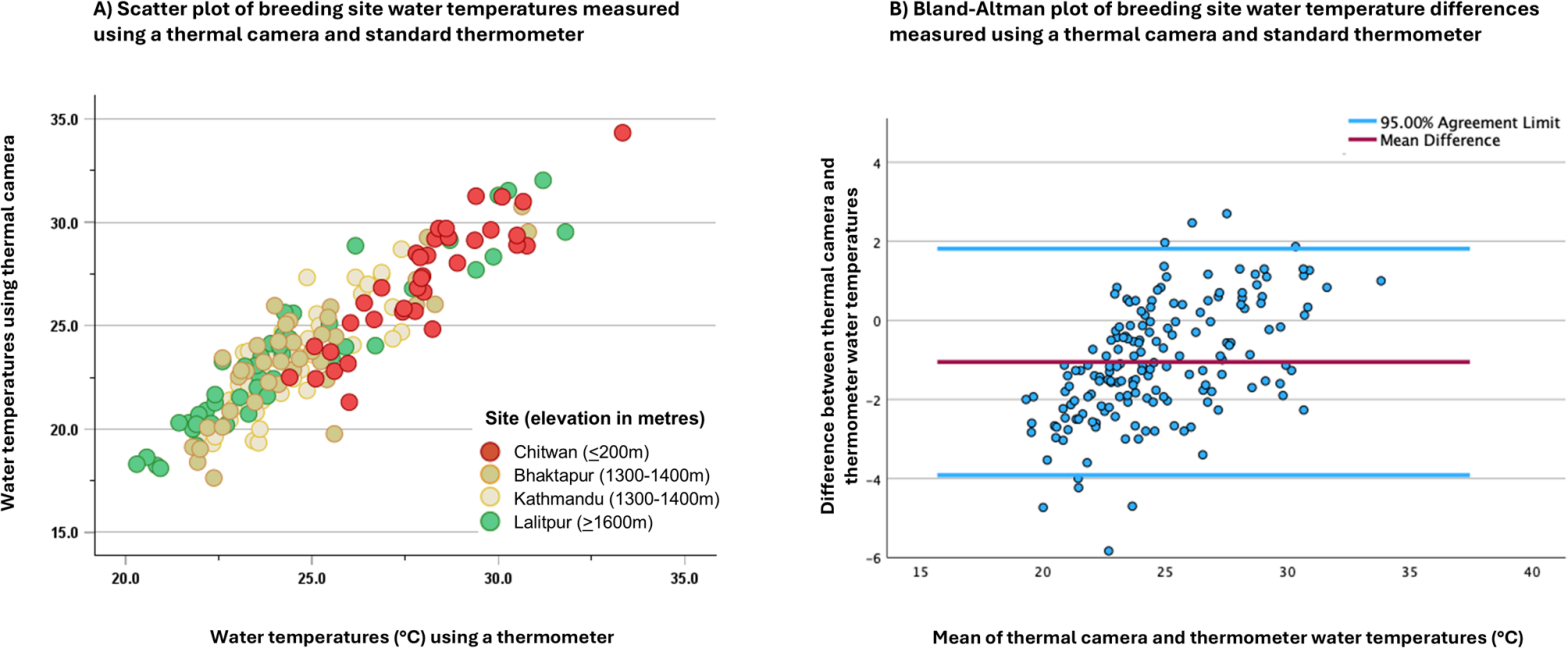
Correlations and agreement between water temperatures of all potential breeding sites measured by a thermal camera and standard thermometer. Note: Graph B. The Bland–Altman plots, the red line represents the mean difference, and the blue upper and lower lines represent the 95% limits of agreement (LoA).

Pearson’s correlation coefficient (r) found a significant positive relationship between the breeding site water temperatures measured using a thermal camera and those measured using a standard thermometer (r = 0.915, *p* < 0.001). Figure 3A scatter plot visually shows the correlations between temperatures. Study locations are delineated by colour to highlight the higher (warmer) temperatures in Chitwan, Terai region and the lower (cooler) temperatures in Lalitpur, Mountain region. In addition, Pearson’s correlation coefficient (r) found a significant positive relationship between the breeding site thermal water temperatures and dissolved oxygen (r = 0.254, *p* < 0.001), but not the other physicochemical parameters including total dissolved solids (TDS), pH, or salinity.

Bland–Altman analysis found a mean temperature difference of -1.05 indicating a negative systematic bias between the two tools. The bias was statistically significant (*p* < 0.001), suggesting that the thermal camera temperatures (mean = 24.2, SD = 0.3) were consistently lower than standard thermometer temperatures (mean = 25.2, SD = 0.2). Figure 3B Bland–Altman plot shows broad agreement with most of the data points (i.e., only 5 outliers) within the 95% limits of agreement (range -3.909 to 1.803), although the limits were relatively wide indicating variability between the tools. Furthermore, an upward trend of the data points indicated a proportional bias, with a statistically significant association (*p* < 0.001), i.e., the differences increased as the temperatures increased.

### Objective 3

#### Breeding site characteristics

Of the 171 potential breeding sites identified, all were made from artificial materials except for one tree breeding site in Lalitpur. Table 3 shows that the majority of breeding sites were made from plastic (63.2% n = 108/171), dark coloured (61.4%,105/171), small to medium in size (35.7–39.8%, n = 61–68/171), had ≤ 50% debris on the water (69.0%, n = 118/171), and were exposed to > 50% sunlight (61.7%, n = 107/171). Broadly, similar trends were observed across the four locations, however, there were significant differences for breeding site material and size (*p* < 0.001, Fisher-Freeman-Halton test), container colour (*p* = 0.016, Fisher’s exact), and exposure to sunlight (*p* < 0.001, Fisher’s exact). Details are available in the supplementary file 1. Notably, compared with the other locations, Bhaktapur had a lower proportion of plastic (39.5%), and dark coloured (39.5%) breeding sites; Chitwan had a lower proportion of large (2.8%) and higher proportion of rubber tyre (19.4%) breeding sites; and Lalitpur had a higher proportion of breeding sites exposed to > 50% sunlight (87.2%).

**Table 3:** Summary of water temperatures by breeding site characteristics across the four study locations.

Ecological zone and study location	Terai Chitwan (urban) ≤ 200 m		Mid-Hills Kathmandu (urban) 1300-1400 m		Mid-Hills Bhaktapur (rural) 1300-1400 m		Mountain Lalitpur (rural) > 1600 m		Overall	
Mean temperatures (temp)	Mean temp (SE)	No. of sites	Mean temp (SE)	No. of sites	Mean temp (SE)	No. of sites	Mean temp (SE)	No. Of sites	Mean temp (SE)	No. of sites
Breeding site characteristics										
Material										
Plastic	27.8 (0.5)	25	23.5 (0.4)	40	23.6 (0.8)	15	24.0 (0.7)	28	24.6 (0.3)^a^	108
Metal/tin	28.3 (3.0)	2	23.9 (2.0)	2	24.7 (0.9)	11	23.6 (1.4)	8	24.6 (0.7)	23
Rubber tyre	25.2 (1.1)	7	19.9 (0.3)	4	23.4 (0.6)	3	18.4 (0.1)	3	22.4 (0.8)	17
Other	25.5 (1.8)	2	23.4 (0.4)	4	21.8 (1.0)	9	22.8 (1.0)	8	22.7 (0.6)	23
Anova p-values	p = 0.168		p = 0.055		p = 0.183		p = 0.072		p = 0.012*	
Colour										
Dark	27.1 (0.7)	23	23.5 (0.5)	34	22.0 (0.6)	15	23.3 (0.6)	33	24.0 (0.3)	105
Light	27.3 (0.7)	13	22.7 (0.6)	16	24.4 (0.6)	23	23.4 (1.2)	14	24.4 (0.4)	66
Anova p-value	p= 0.852		p = 0.303		p = 0.014*		p = 0.905		p = 0.499	
Size										
Small	27.3 (0.7)	20	22.5 (0.5)	10	24.4 (0.5)	18	24.6 (1.2)	13	25.1 (0.5)^c^	61
Medium	26.9 (0.7)	15	22.5 (0.5)	21	22.8 (0.6)	15	23.0 (0.9)	17	23.7 (0.4)	68
Large	29.1 (—)	1	24.3 (0.7)^b^	19	22.2 (1.5)	5	22.8 (0.7)	17	23.6 (0.5)	42
Anova p-value	p = 0.733		p = 0.042*		p = 0.163		p = 0.332		p = 0.023*	
Debris										
Debris ≤ 50%	27.1 (0.6)	25	23.5 (0.4)	38	24.0 (0.6)	24	23.4 (0.7)	31	24.4 (0.3)	118
Debris > 50%	27.3 (0.8)	11	22.4	12	22.6 (0.7)	14	23.2 (0.8)	16	23.7 (0.4)	53
Anova p-value	p = 0.920		p = 0.193		p = 0.146		p = 0.864		p = 0.245	
Sunlight										
Sunlight ≤ 50%	25.7 (0.8)	14	22.1 (0.3)	29	23.6 (0.3)	15	21.6 (0.6)	6	23.2 (0.3)	64
Sunlight > 50%	28.1 (0.6)	22	24.7 (0.6)	21	23.4 (0.8)	23	23.6 (0.6)	41	24.7 (0.4)	107
Anova	p = 0.017*		p < 0.001**		p = 0.848		p = 0.202		p = 0.005*	

* Significant at the 0.05 level.

** Significant at the 0.01 level.

Tukey HSD results.

a. Plastic breeding site water temperatures significantly higher than rubber (p = 0.046) and other (p = 0.05).

b. Large breeding site water temperatures not significantly higher than medium sized breeding sites (p = 0.055).

c. Small breeding site water temperatures significantly higher than medium (p = 0.029) and large (p = 0.047) breeding sites.

#### Breeding site characteristics and their infrared thermal water temperature parameters

The average water temperatures varied by the breeding site characteristics as shown in Table 3. Overall, a one-way ANOVA showed significant temperature differences in the type of breeding site material (*p* = 0.012), its size (*p* = 0.023) and the level of exposure to sunlight (*p* = 0.005).Tukey HSD post hoc comparisons of material type found that breeding sites made of plastic (24.6 °C) had significantly higher temperatures than those made of rubber (22.5 °C; *p* = 0.046) or other materials (22.8 °C; *p* = 0.050). Post hoc comparisons of size found that small breeding sites (25.2 °C) had significantly higher temperatures than medium (23.7 °C; *p* = 0.029) or large (23.6 °C, *p* = 0.047). See supplementary file 1 for more detailed post hoc results.

In terms of sunlight exposure, breeding sites exposed to > 50% sunlight had significantly higher temperatures (24.7 °C) than those exposed to ≤ 50% sunlight (23.2 °C; *p* = 0.005). The trends were similar across the four locations, and significant differences were also found in Chitwan (*p* = 0.017) and Kathmandu (*p* = 0.001).

In Bhaktapur, a one-way ANOVA showed that light coloured breeding sites had significantly higher temperatures than dark coloured breeding sites (*p* = 0.014). In Kathmandu, a one-way ANOVA showed significant temperature differences in the size of breeding site (*p* = 0.042). Post hoc comparisons did not find a significant difference (*p* = 0.055).

The model Type III test indicated that breeding site water temperature was significantly associated with location (F = 7.009, *p* < 0.001), suggesting an overall effect across locations. However, none of the individual fixed effect estimates were statistically significant, which may be due to limited statistical power related to the small sample size, reducing the ability to detect differences between locations. The model Type III test found no significant effects of the breeding site material (F = 1.154, *p* = 0.334), colour (F = 0.036, *p* = 0.851), size (F = 0.478, *p* = 0.622), level of debris (F = 1.456, *p* = 0.232), or exposure to sunlight (F = 1.298, *p* = 0.258). There was also no evidence of a significant interaction between characteristics. Additional information on the model outputs, including fixed effect estimates (i.e. coefficients (β)) and confidence intervals, is available in Supplementary file 1.

### Objective 4

#### Mosquito species and their breeding site characteristics

Of the 171 potential breeding sites identified, 145 were found to have mosquito larvae present at the time of the survey and 136 could be identified to genera (i.e., *Aedes* or *Culex* spp.) or species level.

The main species identified were *Ae. aegypti* (n = 41)*, Ae. albopictus* (n = 29) *and Culex quinquefasciatus* (genus *Culex*; n = 22). Four sites had both *Ae. aegypti* and *Ae. albopictus*, three in Chitwan and one in Bhaktapur. The remaining breeding sites contained a mix of other species from different genera, including *Aedes (Aedes lineatopennis*, *Aedes pseudotaeniatus), Armigeres (Armigeres subalbatus, Armigeres aureolineatus), Culex (Culex pipiens, Culex vishnui, Culex tritaeniorhynchus, Culex fuscocephala), and Anopheles (Anopheles culicifacies)* (Supplementary information, file 2 for details).

*Ae. aegypti* mosquitoes were found in 41 of the 145 breeding sites with mosquito larvae/pupae, the majority in Kathmandu (n = 24/41). None were found in Lalitpur (Table 4). Statistically significant differences were found between *Ae. aegypti* breeding sites and the study locations (*p* < 0.001, Fisher-Freeman-Halton test) and in sites with ≤ 50% debris present (*p* = 0.008, Fisher’s exact) and ≤ 50% sunlight exposure (*p* = 0.002, Fisher’s exact).

**Table 4:** Summary of *Aedes* species presence by the four study locations and breeding site characteristics.

Study location and breeding site characteristics	*Ae. aegypti*		p-value	*Ae. albopictus*		p-value
	Absent n (%)	Present n (%)		Absent n (%)	Present n (%)	
Study location						
Chitwan	21 (61.8)	13 (38.2)	p < 0.001**	22 (64.7)	12 (35.2)	p = 0.035*
Kathmandu	18 (42.9)	24 (57.1)		38 (90.5)	4 (9.5)	
Bhaktapur	25 (86.2)	4 (13.8)		22 (75.9)	7 (24.1)	
Lalitpur	40 (100)	0 (0)		34 (85.0)	6 (15.0)	
Material						
Plastic	67 (70.5)	28 (29.5)	p = 0.146	77 (81.1)	18 (18.9)	p = 0.069
Metal/tin	14 (82.4)	3 (17.6)		15 (88.2)	2 (11.8)	
Rubber tyre	7 (50.0)	7 (50.0)		13 (92.9)	1 (7.1)	
Other	16 (84.2)	3 (15.8)		11 (57.9)	8 (42.1)	
Colour						
Dark	63 (67.0)	31 (33.0)	p = 0.088	75 (79.8)	19 (20.2)	p = 0.931
Light	41 (80.4)	10 (19.6)		41 (80.4)	10 (19.6)	
Size						
Small	35 (70.0)	15 (30.0)	p = 0.648	34 (68.0)	16 (32.0)	p = 0.044*
Medium	41 (69.5)	18 (30.5)		51 (86.4)	8 (13.6)	
Large	28 (77.8)	8 (22.2)		31 (86.1)	5 (13.9)	
Debris						
< 50%	62 (64.6)	34 (35.4)	p = 0.008*	78 (81.3)	18 (18.8)	p = 0.598
> 50%	42 (85.7)	7 (14.3)		38 (77.6)	11 (22.4)	
Sunlight						
< 50%	32 (57.1)	24 (42.9)	p = 0.002*	44 (78.6)	12 (21.4)	p = 0.733
> 50%	72 (80.9)	17 (19.1)		72 (80.9)	17 (19.1)	

*Significant at the 0.05 level.

**Significant at the 0.001 level.

Fisher’s exact test was used for 2 × 2 tables and the Fisher-Freeman-Halton test for 2 × 3 tables.

*Ae. albopictus* mosquitoes were found in 29 of the 145 breeding sites, the majority in Chitwan (n = 12/29) (Table 4). Statistically significant differences were found between *Ae. albopictus* breeding sites and the study locations (*p* = 0.035, Fisher-Freeman-Halton test), and the size (*p* = 0.044, Fisher-Freeman-Halton test), with the highest proportion of *Ae. albopictus* in small breeding sites.

### Objective 5

#### *Ae. aegypti* and *Ae. albopictus* breeding site infrared thermal water temperature parameters by location (ecological region)

The mean breeding site water temperatures for *Ae. aegypti* and *Ae. albopictus* across study locations were found to be significantly different between locations (*p* < 0.001 and p = 0.023 respectively, one-way ANOVA). Data are available in Supplementary file 1and noted here.

Mean temperatures of *Ae. aegypti* breeding sites were around 4.5 °C higher in Chitwan, Terai region (27.5 °C, SE 0.9) than in Kathmandu (23.3 °C, SE 0.5) and Bhaktapur (23.4 °C, SE 1.7), Mid-Hill region. Tukey HSD post hoc comparisons found that Chitwan had significantly higher water temperatures in *Ae. aegypti* breeding sites than Kathmandu (*p* < 0.001), and Bhaktapur (*p* = 0.045).

Similar trends were observed for *Ae. albopictus* with breeding sites temperatures around 4.5 °C higher in Chitwan, Terai region (27.0 °C, SE 0.7) than in Kathmandu (22.4 °C, SE 1.0) and Bhaktapur (22.9 °C, SE 1.4), Mid-Hill region. However, they were only 1 °C higher than in Lalitpur (26.0 °C, SE 1.6). Tukey HSD post hoc comparisons found that Chitwan had significantly higher water temperatures in *Ae. albopictus* breeding sites than Bhaktapur (*p* = 0.048).

#### *Ae. aegypti* and *Ae. albopictus* breeding site infrared thermal water temperature by different times of the day

Exploratory analysis of breeding site water temperatures at different times of the day (morning and midday periods) were based on *Aedes* data collected from 15 households. Four households with seven *Ae. aegypti* breeding sites and five households with five *Ae. albopictus* breeding sites were assessed. A summary of the breeding site characteristics and temperature ranges are presented in Table 5. The largest temperature change (+ 10.4 °C) was in an *Ae. albopictus* small white plastic breeding site with low level of debris and high level of sunlight exposure in Chitwan. The smallest temperature change (+ 0.2 °C) was in *Ae. aegypti* black rubber tyre breeding site with low level of debris and low level of sunlight exposure.

**Table 5:** Summary of selected *Ae. aegypti* and *Ae. albopictus* breeding site temperatures at different times of the day across the four study locations.

Study location and *Aedes* species	Site code	Breeding site characteristics	Morning temp °C	Midday temp °C	Difference in temp °C
Chitwan					
*Ae. aegypti*	CH1C1	Rubber, medium, black, debris (≤ 50%), sunlight (> 50%)	22.5	28.4	+ 5.9
*Ae. albopictus*	CH15C1	Plastic, small, white, debris (≤ 50%), sunlight (> 50%)	25.8	36.2	+ 10.4
Kathmandu					
*Ae. aegypti*	AH1C1	Rubber, medium, black, debris (≤ 50%), sunlight (≤ 50%)	19.7	17.6	-2.1
*Ae. aegypti*	AH1C2	Rubber, medium, black, debris (≤ 50%), sunlight (≤ 50%)	19.7	19.9	+ 0.2
*Ae. aegypti*	AH1C3	Rubber, medium, black, debris (≤ 50%), sunlight (≤ 50%)	19.4	23.4	+ 4.0
*Ae. aegypti*	AH19C1	Plastic, large, blue, debris (> 50%), sunlight (≤ 50%)	20.8	28.9	+ 8.1
*Ae. aegypti*	AH19C2	Plastic, small, white, debris (> 50%), sunlight (≤ 50%)	21.0	29.2	+ 8.2
*Ae. aegypti*	AH22C1	Plastic, large, black, debris (≤ 50%), sunlight (> 50%)	28.7	34.1	+ 5.4
*Ae. albopictus*	AH6C2	Plastic, large, black, debris (≤ 50%), sunlight (> 50%)	19.6	25.6	+ 6.0
Bhaktapur					
*Ae. albopictus*	BH9C1	Clay, medium, brown, debris (> 50%), sunlight (> 50%)	18.4	19.6	+ 1.2
Lalitpur					
*Ae. albopictus*	DH11C2	Rubber, small, black, debris (≤ 50%), sunlight (≤ 50%)	21.6	25.6	+ 4.0
*Ae. albopictus*	DH12C1	Tree, small, green, debris (≤ 50%), sunlight (≤ 50%)	23.1	24.5	+ 1.4

### Objective 6. Case studies of infrared thermal temperatures across different microclimates

**Fig. 4:**
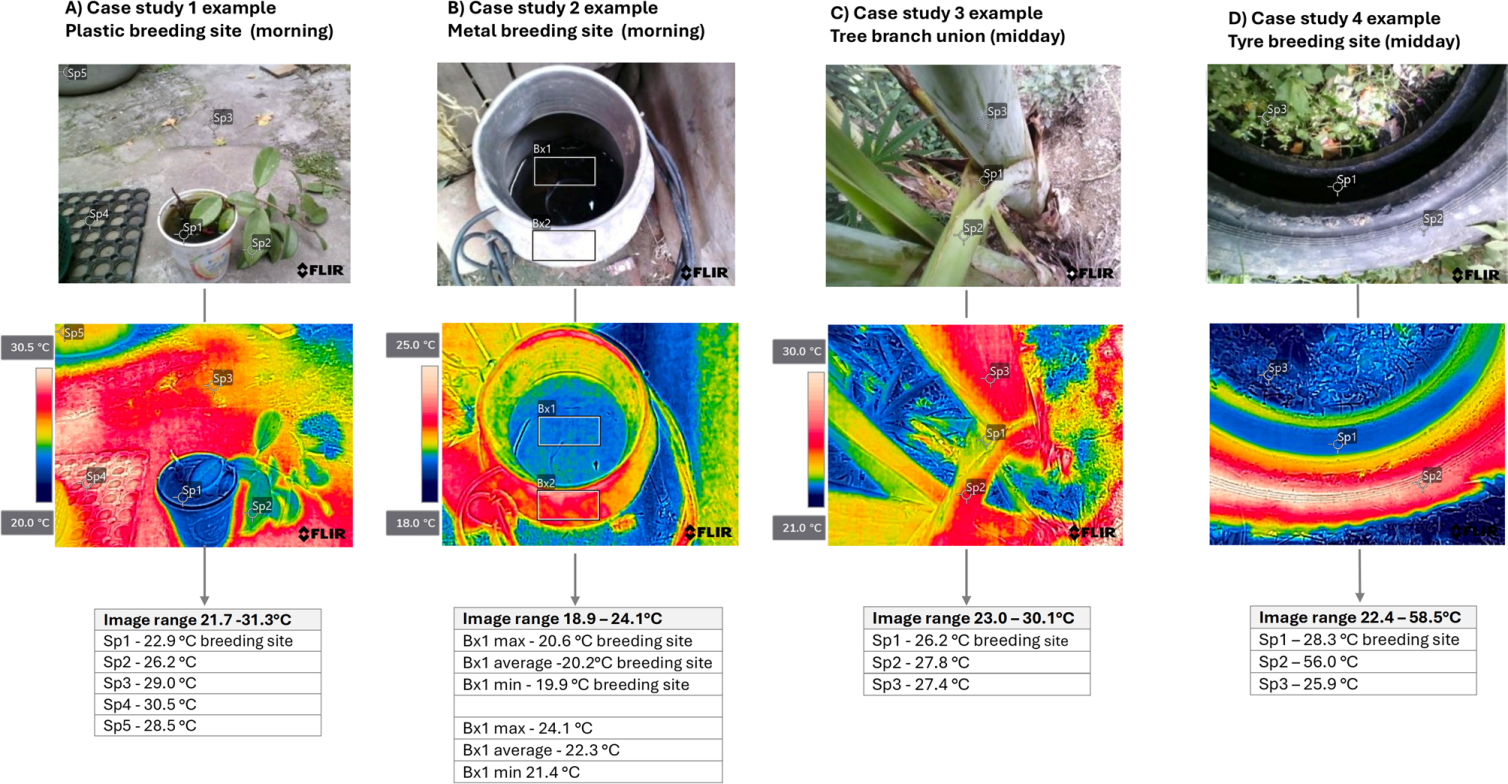
Case study examples of Infrared thermal imaging across different vector breeding sites.

Five case studies were identified from the infrared thermal images collected during the field work that characterised a variety of *Aedes* vector microclimates, visually capturing temperatures of breeding sites and the immediate surroundings. The case studies present examples of i) each location ii) different breeding site materials and ii) different times of the day. A summary of each case study is presented here, with example images presented in Figure 4A-D. A full description of each case study and all images are provided in Supplementary file 3.

- *Case study 1. Plastic water containers (morning)*: Mid**-**Hill Region; Kathmandu District; Urban. Yard. Two containers were identified as *Ae. aegypti* breeding sites (blue plastic container, white plastic plant pot) with temperatures between 1.0- 7.7 °C cooler than other selected spots in the immediate surrounding surfaces (e.g. concrete ground). Figure 4A shows a close up plant pot example, visually highlighting the cooler temperature of the breeding site (Sp1 22.9 °C) compared with the ground including a rubber mat and concrete (Sp3 26.2 °C and Sp4 30.5 °C, respectively).
- *Case study 2. Metal water container (morning)*: Mid**-**Hill Region; Bhaktapur District; Rural. One medium sized metal/tin container was identified as an *Ae. albopictus* and *Cx*. *quinquefasciatus* breeding site. Two areas on the container were compared using a rectangle or Box (Bx) with maximum, minimum and average temperatures presented. Figure 4B highlights the cooler inner water temperatures (Bx1 maximum 20.6 °C) compared to the external part of the container (Bx1 maximum 24.1 °C).
- *Case study 3. Tree water container (morning and midday)*: Mountain (Lower) Region; Lalitpur District; Rural. A tree branch union (i.e. branch joins the trunk) was identified as an *Ae. albopictus* breeding site in a household garden and found to have cooler temperatures than the branches. Comparisons were made between the morning and midday periods and showed an overall increase in temperature of between 3.1 and 4.1 °C in three spots. Figure 4C shows the midday image and highlights the cooler breeding site temperature (Sp1 26.2 °C) compared to the tree branch and trunk (Sp2 27.8 °C and Sp3 27.4 °C, respectively).
- *Case study 4. Rubber water container (morning and midday*): Mountain (Lower) Region; Lalitpur District; Rural. One rubber tyre among four was identified as an *Ae. lineatopennis* breeding site. The whole tyre and the inner and external part of the tyre were examined and temperatures compared between the morning and midday periods using spots (Sp). In the morning the inner tyre breeding site temperature of 19.6 °C was 4.1 °C cooler than the external tyre spot (Sp2, 23.7 °C). Figure 4D shows the midday image, highlighting the large differences in temperature between the inner breeding site (Sp1, 28.3 °C) and the external part of the tyre (Sp2, 56.0 °C).
- *Case study 5. Rubber water container (morning and midday)*: Terai Region; Chitwan District; Urban. One rubber tyre was identified as an *Ae. aegypti* breeding site. The inner and external part of the tyre were examined and temperatures compared between the morning and midday periods using a rectangle or Box (Bx). In the morning the inner tyre breeding site temperature ranges were between 0.9 and 1.4 °C cooler than the external tyre ranges. At midday, the inner tyre breeding site temperature increased to a maximum of 31.1 °C, which was between 14.1 and 15.0 °C cooler than the external tyre temperature ranges (Bx maximum 45.2 °C). Images are available in the Supplementary file 3.

## Discussion

This pilot feasibility study is the first to explore mosquito vector habitats using an infrared thermal camera as a novel tool to assess temperature parameters across different types of breeding sites, ecological regions, and times of day. We focused on *Aedes* mosquitoes, which primarily breed in containers in peri-/domestic environments, but also identified a range of different mosquito species exploiting similar habitats, as shown in recent studies^16^. These species could be explored further, particularly those that are vectors of public health concern. For example, C*x. quinquefasciatus* is a major vector of lymphatic filariasis^16,55,56^. This species has been implicated in Japanese encephalitis elsewhere and may act as a potential vector alongside the primary vector *Cx. tritaeniorhynchus* ^57–60^.

A weak positive correlation was found between aquatic mosquito habitat temperatures and dissolved oxygen, which differs from the expected negative relationship. This trend may be influenced by the steep altitudinal gradient in Nepal ^61,62^ as oxygen solubility in water declines with decreasing atmospheric pressure, which may be important at high altitudes ^63^. However, notable differences in dissolved oxygen levels were found between the mid-Hill locations of Bhaktapur and Kathmandu, and Bhaktapur was higher than Chitwan in the lowland Terai region. These differences may be related to rural and urban differences, or each location’s unique composition of breeding site characteristics (e.g., Bhaktapur had lower proportions of plastic and dark sites than these other locations). Physicochemical parameters of water, including temperature, have also been shown to vary by altitude in Nepal ^62^. Further investigation is warranted into the role of key breeding site characteristics, environmental conditions, and the potential impact of altitude, particularly in mountain aquatic systems. As temperature does not act in isolation, additional exploration of interacting factors such as humidity, resource availability, larval density, competition, habitat characteristics, and geographic origin is needed ^37–39^.

We found that infrared thermal temperatures were broadly in agreement with those obtained using a standard thermometer, however, it did consistently show a 1 °C lower reading and divergence at higher temperatures. The lower readings may be due to the differences between surface temperatures and subsurface temperatures, which tend to be more stable ^64^. Surface water temperatures may respond more to sunlight exposure and air temperature. The differences may also be related to the emissivity setting in the thermal camera, which was set at 0.9 to account for different surface types around the breeding sites, but was a little low for water (~ 0.92–0.98) and may have led to an underestimate. We show that Infrared imaging is promising for field characterisation and visualisation of thermal heterogeneity, but its quantitative use will require calibration, attention to emissivity, water-surface versus subsurface differences, sunlight exposure, and uncertainty analysis.

Notwithstanding these limitations and considerations, the use of thermal imaging has the potential to be developed further for the characterisation of vector breeding sites, but will require more calibration and uncertainty analysis addressing emissivity and environmental factors. Thermal imaging may serve as a practical and innovative tool for research and programmatic purposes; for example, it could be used to assess varying temperatures in complex urban and rural microclimates; or evaluate seasonal thermal patterns of vector habitats and physicochemical parameters across different ecological regions and altitudes^30,34^. The overall model outputs suggest that location and corresponding ecological zones had a significant effect on temperature, whereas the breeding site characteristics (material, colour, size, debris, sunlight) did not show evidence of an independent effect once location was accounted for. The lack of statistically significant individual fixed effect estimates may be related to how the model defines comparisons between groups and the choice of reference category, as well as limited statistical power associated with the small sample size. Further research with larger, more diverse samples is needed to validate these results and calibrate the thermal camera usage across different settings.

Overall, we found that breeding site temperatures were comparable to those reported in other studies^34–36^, however, there were clear differences between ecological regions. The warm temperatures in the lowland Terai region align with the high dengue risk^1^, and vector occurrence in previous research^10^. Aquatic mosquito habitat temperatures in Chitwan were mostly within the optimal range, but some were near to, or exceeded the upper limit of 32 °C as found in other studies^33,36^. Infrared thermal imaging may be an effective tool for monitoring *Aedes* species in this high-risk dengue region, as studies have shown that temperatures over 35 °C could slow, impede, or stop larval development^33,65^. This may be particularly useful for Chitwan breeding sites made of plastic or metal/tin, which had high mean temperatures of 27.8 °C and 28.3 °C, respectively.

In the Mid-Hill and Mountain regions, the mosquito aquatic temperatures were also mostly within the optimal range, albeit lower, with some below the lower limit or < 20 °C. These observations are consistent with the possibility that warmer peri-domestic microhabitats may facilitate *Aedes* persistence in cooler, higher-altitude settings, but behavioural selection and local adaptation were not tested in this study^66–69^. We found that most breeding sites in Lalitpur (87.2%) had > 50% sunlight exposure compared with the other locations (42.0–61.1%). This may help explain the presence of *Ae. albopictus* in this mountain region, where we found one small dark plastic breeding site in full sun with water temperatures over 30 °C.

Our case studies, including plastic plant pots, metal water containers, tree holes, and discarded rubber tyres, provide some insight into the microclimates of vectors i.e. mosquito breeding site and immediate surrounding area. Overall, plastic containers were the most abundant type of breeding sites, recorded higher temperatures across all ecological regions, and showed one of the highest daily temperature fluctuations, supporting findings from other studies^70^. Our exploratory analysis of aquatic breeding habitats at different times of the day was limited to 15 households. Future infrared thermal imaging research focusing on temperature fluctuation of plastic breeding sites and how these influence abiotic conditions may help determine their impact on transmission. Nepal already recognizes the challenges of plastic to the environment, and the lack of effective recycling is a major problem that potentially could increase and/or sustain the risk of dengue^71,72^.

The widespread presence of artificial containers, including rubber tyres, highlights the growing impact of human activity and risk of dengue. International trade in second-hand tyres has been linked to the global spread of dengue^73^. Field studies in Nepal show that discarded tyres are major breeding sites that create conducive habitats for *Aedes* vectors^10,19^. Our case study visually shows that the rubber tyre inner aquatic temperatures were within the optimal range at different times of the day (i.e., no major fluctuations), compared with the tyre external temperature, which increased by > 23 °C and exceeded 50 °C in the midday period. Rubber is a poor conductor of heat and the external part of the tyre may add a thermal barrier, which provides a conducive inner aquatic environment for *Aedes* species to exploit; hence why this type of breeding site is likely to have facilitated the global expansion of dengue.

The main limitation to this pilot study was the small sample size and cross-sectional approach, constrained by seed funding. A key strength, however, is that it highlights a portable tool that could advance our knowledge of the thermal ecology of vectors and vector-borne diseases. The thermal camera effectively captured mosquito breeding site temperatures on-the-spot during fieldwork, while thermal images helped to characterise the vector microclimates, by capturing temperatures of both the breeding site and its surroundings, thereby revealing micro-scale heterogeneity that vectors may exploit for breeding. Systematic collection and collation of digital thermal data and images could be coupled with high resolution geospatial and environmental mapping and modelling to better understand how temperature influences the transmission of dengue and other vector-borne disease at macro- and micro-scales.

## Supplementary Information

Below is the link to the electronic supplementary material.

Supplementary Material 1

Supplementary Material 2

Supplementary Material 3

## Data Availability

The data supporting the findings of this study are included in the paper and the supplementary information. Additional survey data or images are available from the corresponding authors upon reasonable request.
