# Supplementary material for: I﻿nfrared thermal imaging to determine temperature parameters of dengue vector habitats across ecological regions of Nepal: a pilot feasibility study
Source: Sci Rep. 2026 Jul 27;16:23328. doi: 10.1038/s41598-026-61694-1 (PMC13408821; doi:10.1038/s41598-026-61694-1)
Supplement: Supplementary file 1 — Supplementary Material 1 [file 41598_2026_61694_MOESM1_ESM.docx]

**Supplementary tables and figures for each objective**

**Objective 1: Ambient environmental conditions and mosquito breeding site water parameters**

S1 Table - Post hoc results using the Tukey HSD test for differences between locations for household level ambient temperature and humidity and mosquito breeding site water temperature and dissolved oxygen measurements (SPSS output)

| **Multiple Comparisons** | | | | | | | |
| --- | --- | --- | --- | --- | --- | --- | --- |
| Tukey HSD | | | | | | | |
| Dependent Variable | (I) Location | (J) Location | Mean Difference (I-J) | Std. Error | Sig. | 95% Confidence Interval | |
|  |  |  |  |  |  | Lower Bound | Upper Bound |
| Ambient temperature | Chitwan | Kathmandu | 1.85^*^ | .50 | .002 | .54 | 3.16 |
|  |  | Bhaktapur | 1.70^*^ | .54 | .010 | .31 | 3.09 |
|  |  | Lalitpur | 1.03 | .51 | .183 | -.29 | 2.36 |
|  | Kathmandu | Chitwan | -1.85^*^ | .50 | .002 | -3.16 | -.54 |
|  |  | Bhaktapur | -.15 | .50 | .990 | -1.44 | 1.13 |
|  |  | Lalitpur | -.82 | .47 | .304 | -2.03 | .40 |
|  | Bhaktapur | Chitwan | -1.70^*^ | .54 | .010 | -3.09 | -.31 |
|  |  | Kathmandu | .15 | .50 | .990 | -1.13 | 1.44 |
|  |  | Lalitpur | -.66 | .50 | .552 | -1.97 | .64 |
|  | Lalitpur | Chitwan | -1.03 | .51 | .183 | -2.36 | .29 |
|  |  | Kathmandu | .82 | .47 | .304 | -.40 | 2.03 |
|  |  | Bhaktapur | .66 | .50 | .552 | -.64 | 1.97 |
| Ambient humidity | Chitwan | Kathmandu | 17.63^*^ | 1.7 | <.001 | 13.12 | 22.15 |
|  |  | Bhaktapur | 9.46^*^ | 1.8 | <.001 | 4.66 | 14.26 |
|  |  | Lalitpur | 17.59^*^ | 1.8 | <.001 | 13.02 | 22.16 |
|  | Kathmandu | Chitwan | -17.63^*^ | 1.7 | <.001 | -22.15 | -13.12 |
|  |  | Bhaktapur | -8.18^*^ | 1.7 | <.001 | -12.62 | -3.74 |
|  |  | Lalitpur | -.05 | 1.6 | 1.000 | -4.24 | 4.15 |
|  | Bhaktapur | Chitwan | -9.46^*^ | 1.8 | <.001 | -14.26 | -4.66 |
|  |  | Kathmandu | 8.18^*^ | 1.7 | <.001 | 3.74 | 12.62 |
|  |  | Lalitpur | 8.13^*^ | 1.7 | <.001 | 3.63 | 12.63 |
|  | Lalitpur | Chitwan | -17.59^*^ | 1.8 | <.001 | -22.16 | -13.02 |
|  |  | Kathmandu | .05 | 1.6 | 1.000 | -4.15 | 4.24 |
|  |  | Bhaktapur | -8.13^*^ | 1.7 | <.001 | -12.63 | -3.63 |
| Thermal camera temperature | Chitwan | Kathmandu | 3.97^*^ | .67 | <.001 | 2.24 | 5.70 |
|  |  | Bhaktapur | 3.69^*^ | .71 | <.001 | 1.85 | 5.53 |
|  |  | Lalitpur | 3.81^*^ | .68 | <.001 | 2.05 | 5.56 |
|  | Kathmandu | Chitwan | -3.97^*^ | .67 | <.001 | -5.70 | -2.24 |
|  |  | Bhaktapur | -.28 | .66 | .975 | -1.98 | 1.43 |
|  |  | Lalitpur | -.16 | .62 | .994 | -1.77 | 1.45 |
|  | Bhaktapur | Chitwan | -3.69^*^ | .71 | <.001 | -5.53 | -1.85 |
|  |  | Kathmandu | .28 | .66 | .975 | -1.43 | 1.98 |
|  |  | Lalitpur | .11 | .67 | .998 | -1.61 | 1.84 |
|  | Lalitpur | Chitwan | -3.81^*^ | .68 | <.001 | -5.56 | -2.05 |
|  |  | Kathmandu | .16 | .62 | .994 | -1.45 | 1.77 |
|  |  | Bhaktapur | -.11 | .67 | .998 | -1.84 | 1.61 |
| Thermometer temperature | Chitwan | Kathmandu | 3.55^*^ | .49 | <.001 | 2.29 | 4.82 |
|  |  | Bhaktapur | 3.31^*^ | .52 | <.001 | 1.97 | 4.66 |
|  |  | Lalitpur | 3.65^*^ | .49 | <.001 | 2.37 | 4.93 |
|  | Kathmandu | Chitwan | -3.55^*^ | .49 | <.001 | -4.82 | -2.29 |
|  |  | Bhaktapur | -.24 | .48 | .958 | -1.49 | 1.00 |
|  |  | Lalitpur | .10 | .45 | .997 | -1.08 | 1.27 |
|  | Bhaktapur | Chitwan | -3.31^*^ | .52 | <.001 | -4.66 | -1.97 |
|  |  | Kathmandu | .24 | .48 | .958 | -1.00 | 1.49 |
|  |  | Lalitpur | .34 | .49 | .898 | -.92 | 1.60 |
|  | Lalitpur | Chitwan | -3.65^*^ | .49 | <.001 | -4.93 | -2.37 |
|  |  | Kathmandu | -.10 | .45 | .997 | -1.27 | 1.08 |
|  |  | Bhaktapur | -.34 | .49 | .898 | -1.60 | .92 |
| Dissolved oxygen | Chitwan | Kathmandu | .51 | .20 | .058 | -.01 | 1.04 |
|  |  | Bhaktapur | -.26 | .22 | .613 | -.82 | .30 |
|  |  | Lalitpur | .90^*^ | .21 | <.001 | .37 | 1.44 |
|  | Kathmandu | Chitwan | -.51 | .20 | .058 | -1.04 | .01 |
|  |  | Bhaktapur | -.78^*^ | .20 | <.001 | -1.30 | -.26 |
|  |  | Lalitpur | .39 | .19 | .171 | -.10 | .88 |
|  | Bhaktapur | Chitwan | .26 | .22 | .613 | -.30 | .82 |
|  |  | Kathmandu | .78^*^ | .20 | <.001 | .26 | 1.30 |
|  |  | Lalitpur | 1.17^*^ | .20 | <.001 | .64 | 1.69 |
|  | Lalitpur | Chitwan | -.90^*^ | .21 | <.001 | -1.44 | -.37 |
|  |  | Kathmandu | -.39 | .19 | 0.171 | -.88 | .10 |
|  |  | Bhaktapur | -1.17^*^ | .20 | <.001 | -1.69 | -.64 |
| *. The mean difference is significant at the 0.05 level. | | | | | | | |

**Objective 3. Breeding site characteristics and water temperatures by characteristics**

S2 Table – Summary of breeding site characteristics by the four study locations

| **Ecological zone and study location**  No. of sites | **Terai**  **Chitwan (urban)**  **<200 m**  n=36 | **Mid-Hills**  **Kathmandu**  **(urban)**  **1300-1400 m**  n=50 | **Mid-Hills**  **Bhaktapur**  **(rural)**  **1300-1400 m**  n=38 | **Mountain**  **Lalitpur**  **(rural)**  **>1600 m**  n=47 | **Overall**  n=171 | **p-value** |
| --- | --- | --- | --- | --- | --- | --- |
| **Measure** | **n (%) column** | **n (%) column** | **n (%) column** | **n (%) column** | **n (%) column** |  |
| **Material** |  |  |  |  |  |  |
| Plastic | 25 (69.4) | 40 (80.0) | 15 (39.5) | 28 (59.6) | 108 (63.2) | p<0.001**^ab^ |
| Metal/tin | 2 (5.6) | 2 (4.0) | 11 (28.9) | 8 (17.0) | 23 (13.5) |  |
| Rubber tyre | 7 (19.4) | 4 (8.0) | 3 (7.9) | 3 (6.4) | 17 (9.9) |  |
| Other | 2 (5.6) | 4 (8.0) | 9 (23.7) | 8 (17.0) | 23 (13.5) |  |
|  |  |  |  |  |  |  |
| **Colour** |  |  |  |  |  |  |
| Dark | 23 (63.9) | 34 (68.0) | 15 (39.5) | 33 (70.2) | 105 (61.4) | p=0.016* |
| Light | 13 (36.1) | 16 (32.0) | 23 (60.5) | 14 (29.8) | 66 (38.6) |  |
|  |  |  |  |  |  |  |
| **Size** |  |  |  |  |  |  |
| Small | 20 (55.6) | 10 (20.0) | 18 (47.4) | 13 (27.7) | 61 (35.7) | p<0.001** |
| Medium | 15 (41.7) | 21 (42.0) | 15 (39.5) | 17 (36.2) | 68 (39.8) |  |
| Large | 1 (2.8) | 19 (38.0) | 5 (13.2) | 17 (36.2) | 42 (24.6) |  |
|  |  |  |  |  |  |  |
| **Debris** |  |  |  |  |  |  |
| Debris <50% | 25 (69.4) | 38 (76.0) | 24 (63.2) | 31 (66.0) | 118 (69.0) | p=0.581 |
| Debris >50% | 11 (30.6) | 12 (24.0) | 14 (36.8) | 16 (34.0) | 53 (31.0) |  |
|  |  |  |  |  |  |  |
| **Sunlight** |  |  |  |  |  |  |
| Sunlight <50% | 14 (38.9) | 29 (58.0) | 15 (39.5) | 6 (12.8) | 64 (37.4) | p<0.001** |
| Sunlight >50% | 22 (61.1) | 21 (42.0) | 23 (60.5) | 41 (87.2) | 107 (62.6) |  |

* Significant at the 0.05 level

** Significant at the 0.001 level

Note: Fisher’s exact test was used for 2x4 tables and the Fisher-Freeman-Halton test for 3x4 and 4x4 tables. Due to larger table for material, a Monte Carlos estimate of the exact p=value was used.

| ^a^ 6 cells (37.5%) have expected count less than 5. The minimum expected count is 3.58. |
| --- |
| ^b^ Based on Monte Carlo simulation |

S3 Table – Post hoc results using the Tukey HSD test for overall temperature differences between the different types of breeding sites materials (SPSS output)

| **Multiple Comparisons** | | | | | | |
| --- | --- | --- | --- | --- | --- | --- |
|  | | | | | | |
| Tukey HSD | | | | | | |
| (i) Material | (J) Material | Mean Difference (I-J) | Std. Error | Sig. | 95% Confidence Interval | |
|  |  |  |  |  | Lower Bound | Upper Bound |
| Metal/Tin | Other | 1.83 | .942 | .213 | -.61 | 4.28 |
|  | Plastic | -.07 | .733 | 1.000 | -1.98 | 1.83 |
|  | Rubber tyre | 2.12 | 1.021 | .167 | -.54 | 4.77 |
| Other | Metal/Tin | -1.83 | .942 | .213 | -4.28 | .61 |
|  | Plastic | -1.91^*^ | .733 | .050 | -3.81 | .00 |
|  | Rubber tyre | .28 | 1.021 | .993 | -2.37 | 2.93 |
| Plastic | Metal/Tin | .07 | .733 | 1.000 | -1.83 | 1.98 |
|  | Other | 1.91^*^ | .733 | .050 | .00 | 3.81 |
|  | Rubber tyre | 2.19^*^ | .833 | .046 | .02 | 4.35 |
| Rubber tyre | Metal/Tin | -2.12 | 1.021 | .167 | -4.77 | .54 |
|  | Other | -.28 | 1.021 | .993 | -2.93 | 2.37 |
|  | Plastic | -2.19^*^ | .833 | .046 | -4.35 | -.02 |
| Based on observed means. The error term is Mean Square (Error) = 10.199. | | | | | | |
| *. The mean difference is significant at the .05 level. | | | | | | |

S4 Table – Post hoc results using the Tukey HSD test for overall temperature differences between the different sizes of breeding sites (SPSS output)

| **Multiple Comparisons** | | | | | | |
| --- | --- | --- | --- | --- | --- | --- |
|  | | | | | | |
| Tukey HSD | | | | | | |
| (I) Size | (J) Size | Mean Difference (I-J) | Std. Error | Sig. | 95% Confidence Interval | |
|  |  |  |  |  | Lower Bound | Upper Bound |
| Large | Medium | -.08 | .63 | .991 | -1.56 | 1.40 |
|  | Small | -1.53^*^ | .64 | .047 | -3.05 | -.02 |
| Medium | Large | .08 | .63 | .991 | -1.40 | 1.56 |
|  | Small | -1.45^*^ | .56 | .029 | -2.79 | -.12 |
| Small | Large | 1.53^*^ | .64 | .047 | .02 | 3.05 |
|  | Medium | 1.45^*^ | .56 | .029 | .12 | 2.79 |
| Based on observed means. The error term is Mean Square (Error) = 10.199. | | | | | | |
| *. The mean difference is significant at the .05 level. | | | | | | |

S5 Table – Post hoc results using the Tukey HSD test for Kathmandu temperature differences between the different sizes of breeding sites (SPSS output)

| **Multiple Comparisons** | | | | | | |
| --- | --- | --- | --- | --- | --- | --- |
|  | | | | | | |
| Tukey HSD | | | | | | |
| (I) Size | (J) Size | Mean Difference (I-J) | Std. Error | Sig. | 95% Confidence Interval | |
|  |  |  |  |  | Lower Bound | Upper Bound |
| Large | Medium | 1.82 | .77 | .055 | -.034 | 3.68 |
|  | Small | 1.87 | .95 | .130 | -.422 | 4.16 |
| Medium | Large | -1.82 | .77 | .055 | -3.68 | .03 |
|  | Small | .05 | .93 | .999 | -2.21 | 2.30 |
| Small | Large | -1.87 | .95 | .130 | -4.16 | .42 |
|  | Medium | -.05 | .93 | .999 | -2.30 | 2.21 |

Based on observed means.
The error term is Mean Square (Error) = 5.865

S6 Table – Model Type III Test results for factors influencing breeding site temperatures (SPSS output)

| **Type III Tests of Fixed Effects**^a^ | | | | |
| --- | --- | --- | --- | --- |
| Source | Numerator df | Denominator df | F | Sig. |
| Intercept | 1 | 61.714 | 3533.602 | <.001 |
| Location | 3 | 63.599 | 7.009 | <.001 |
| Material | 3 | 64.558 | 1.154 | .334 |
| Colour | 1 | 76.891 | .036 | .851 |
| Size | 2 | 75.951 | .478 | .622 |
| Debris | 1 | 62.538 | 1.456 | .232 |
| Sunlight | 1 | 76.533 | 1.298 | .258 |

S7 Table - Model estimates of fixed effects results for factors influencing breeding site temperatures (SPSS output)

| **Estimates of Fixed Effects** | | | | | | | |
| --- | --- | --- | --- | --- | --- | --- | --- |
| Parameter | Estimate* | Std. Error | df | t | Sig. | 95% Confidence Interval | |
|  |  |  |  |  |  | Lower Bound | Upper Bound |
| Intercept | 20.925 | 12.577 | 67.571 | 1.664 | .101 | -4.175 | 46.025 |
| Location - Kathmandu | -6.430 | 9.644 | 71.907 | -.667 | .507 | -25.655 | 12.795 |
| Location - Lalitpur | -7.333 | 5.180 | 71.275 | -1.416 | .161 | -17.661 | 2.995 |
| Location - Bhaktapur | 10.959 | 17.878 | 66.161 | .613 | .542 | -24.733 | 46.652 |
| Location - Chitwan | 0^b^ | 0 | . | . | . | . | . |
|  |  |  |  |  |  |  |  |
| Material - Metal/Tin | .494 | 16.649 | 62.576 | .030 | .976 | -32.782 | 33.769 |
| Material - Other | -24.279 | 36.057 | 74.993 | -.673 | .503 | -96.108 | 47.550 |
| Material - Plastic | 6.764 | 12.458 | 67.752 | .543 | .589 | -18.097 | 31.625 |
| Material - Rubber tyre | 0b | 0 | . | . | . | . | . |
|  |  |  |  |  |  |  |  |
| Colour - Dark | 2.409 | 9.420 | 74.467 | .256 | .799 | -16.358 | 21.177 |
| Colour - Light | 0b | 0 | . | . | . | . | . |
|  |  |  |  |  |  |  |  |
| Size - Large | -5.201 | 12.011 | 74.113 | -.433 | .666 | -29.132 | 18.731 |
| Size - Medium | -1.422 | 2.729 | 57.250 | -.521 | .604 | -6.886 | 4.042 |
| Size - Small | 0b | 0 | . | . | . | . | . |
|  |  |  |  |  |  |  |  |
| Debris - <50% | 1.494 | 8.332 | 55.342 | .179 | .858 | -15.201 | 18.190 |
| Debris - > 50% | 0b | 0 | . | . | . | . | . |
|  |  |  |  |  |  |  |  |
| Sunlight - <50% | .990 | 12.971 | 57.916 | .076 | .939 | -24.975 | 26.955 |
| Sunlight - >50% | 0b | 0 | . | . | . | . | . |

Note. Estimate = the **coefficient (β)**

**Objective 5. *Aedes aegypti* and *Aedes albopictus*** **breeding site temperature by location**

S8 Table. Summary of dengue *Aedes* mosquito temperature means and ranges by study location.

| **Ecological Zone and study location** | **Terai**  **Chitwan**  **(urban)**  **200m** | | **Mid-Hills**  **Kathmandu**  **(urban)**  **1300-1400 m** | | **Mid-Hills**  **Bhaktapur**  **(rural)**  **1300-1400 m** | | **Mountain**  **Lalitpur**  **(rural)**  **>1600 m** | | **Overall** | |
| --- | --- | --- | --- | --- | --- | --- | --- | --- | --- | --- |
| **Measures** | **Mean**  **(SE)**  **range** | **No** | **Mean**  **(SE)**  **range** | **No.** | **Mean**  **(SE)**  **range** | **No.** | **Mean**  **(SE)**  **range** | **No** | **Mean**  **(SE)**  **range** | **No.** |
| **Breeding site characteristics** | | | | | | | | | | |
| **Species** |  |  |  |  |  |  |  |  |  |  |
| *Aedes aegypti* | 27.5 (0.9) | 13 | 23.3 (0.5) | 24 | 23.4 (1.7) | 4 |  | 0 | 24.6 (0.5) | 41 |
|  | 22.5-34.3 |  | 19.4-28.7 |  | 19.0-27.2 |  |  |  | 19.0-34.3 |  |
|  |  |  |  |  |  |  |  |  |  |  |
|  |  |  |  |  |  |  |  |  |  |  |
| *Aedes albopictus* | 27.0 (0.7) | 12 | 22.4 (1.0) | 4 | 22.9 (1.4) | 7 | 26.0 (1.6) | 6 | 25.1 (0.7) | 29 |
|  | 22.4 -31.3 |  | 19.6-24.4 |  | 18.4-29.3 |  | 21.6-32.0 |  | 18.4-32.0 |  |
|  |  |  |  |  |  |  |  |  |  |  |

S8 Table - Post hoc results using the Tukey HSD test for differences between locations of *Aedes aegypti* breeding sites

| **Multiple Comparisons** | | | | | | |
| --- | --- | --- | --- | --- | --- | --- |
|  | | | | | | |
| Tukey HSD | | | | | | |
| (i) Location | (J) Location | Mean Difference (I-J) | Std. Error | Sig. | 95% Confidence Interval | |
|  |  |  |  |  | Lower Bound | Upper Bound |
| Kathmandu | Chitwan | -4.3^*^ | 1.0 | <.001 | -6.7 | -1.8 |
|  | Bhaktapur | -.1 | 1.6 | .996 | -4.0 | 3.7 |
| Bhaktapur | Chitwan | -4.1^*^ | 1.7 | .045 | -8.2 | -.1 |
|  | Kathmandu | .1 | 1.6 | .996 | -3.7 | 4.0 |
| Chitwan | Kathmandu | 4.3^*^ | 1.0 | <.001 | 1.8 | 6.7 |
|  | Bhaktapur | 4.1^*^ | 1.7 | .045 | .1 | 8.2 |
| *. The mean difference is significant at the 0.05 level. | | | | | | |

S9 Table - Post hoc results using the Tukey HSD test for differences between locations of *Aedes albopictus* breeding sites

| **Multiple Comparisons** | | | | | | |
| --- | --- | --- | --- | --- | --- | --- |
| Tukey HSD | | | | | | |
| (I) Location | (J) Location | Mean Difference (I-J) | Std. Error | Sig. | 95% Confidence Interval | |
|  |  |  |  |  | Lower Bound | Upper Bound |
| Chitwan | Kathmandu | 4.59 | 1.810 | .0787 | -.39 | 9.57 |
|  | Bhaktapur | 4.13^*^ | 1.491 | .0482 | .03 | 8.23 |
|  | Lalitpur | .98 | 1.568 | .9229 | -3.33 | 5.29 |
| Kathmandu | Chitwan | -4.59 | 1.810 | .0787 | -9.57 | .39 |
|  | Bhaktapur | -.46 | 1.965 | .9953 | -5.87 | 4.95 |
|  | Lalitpur | -3.61 | 2.024 | .3049 | -9.18 | 1.96 |
| Bhaktapur | Chitwan | -4.13^*^ | 1.491 | .0482 | -8.23 | -.03 |
|  | Kathmandu | .46 | 1.965 | .9953 | -4.95 | 5.87 |
|  | Lalitpur | -3.15 | 1.745 | .2950 | -7.95 | 1.65 |
| Lalitpur | Chitwan | -.98 | 1.568 | .9229 | -5.29 | 3.33 |
|  | Kathmandu | 3.61 | 2.024 | .3049 | -1.96 | 9.18 |
|  | Bhaktapur | 3.15 | 1.745 | .2950 | -1.65 | 7.95 |
| *. The mean difference is significant at the 0.05 level. | | | | | | |

S1 Figures A and B. Range of water temperatures of mosquito breeding sites across study locations

1. *Aedes* *aegypti*

*
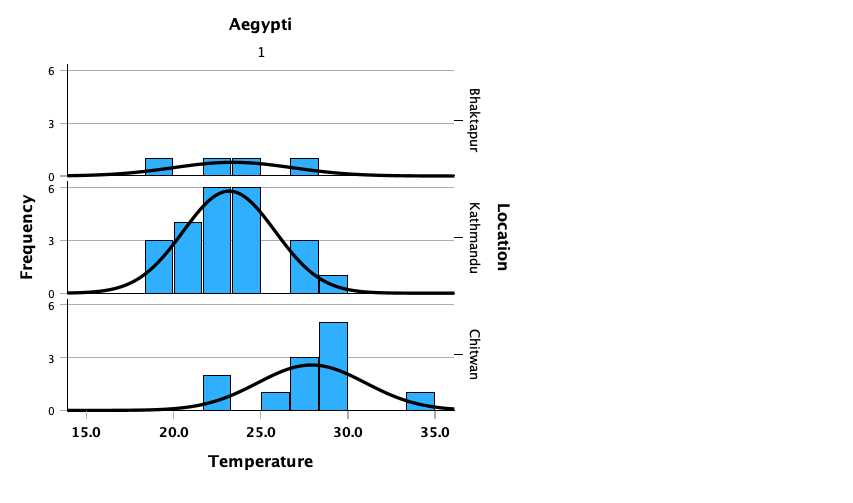
*

1. *Aedes* *albopictus*

*
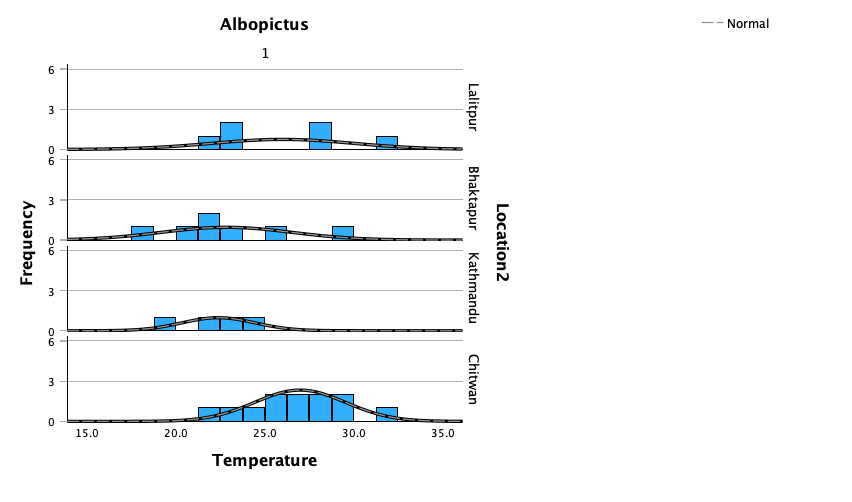
*
