# Supplementary material for: I﻿nfrared thermal imaging to determine temperature parameters of dengue vector habitats across ecological regions of Nepal: a pilot feasibility study
Source: Sci Rep. 2026 Jul 27;16:23328. doi: 10.1038/s41598-026-61694-1 (PMC13408821; doi:10.1038/s41598-026-61694-1)
Supplement: Supplementary file 3 — Supplementary Material 3 [file 41598_2026_61694_MOESM3_ESM.docx]

**Supplementary file 3 Case studies**

**Case study 1. Plastic water containers (morning)**

**Location:** Mid**-**Hill Region; Kathmandu District; Urban

**Dengue vector breeding sites**; Artificial; 1. Plastic; Blue container, medium size, dark colour, <50% debris and <50% sunlight and 2. White pot plant, medium size, light colour, <50% debris and <50% sunlight

**Mosquito larvae/pupae estimates**: Site 1. >300 larvae; breeding site 2 approx. 70

**Mosquito species identified**: *Ae. aegypti* in both breeding sites

**Figure image and breeding and temperature site descriptions**

**A) Multiple containers in a yard** – seven spots compared including five containers (Sp1-Sp5) and two concrete ground spots (Sp6, Sp7). Two containers were identified as mosquito breeding sites (Sp1, Sp2) with temperatures 23.6°C and 22.0°C respectively, which were between 1.0- 7.7°C cooler other spots.

**B) Plastic (blue) breeding site 1** (Sp1) compared with spots in two plastic containers without water (Sp2, Sp3) and two spots on concrete ground (Sp4, Sp5). Breeding site temperature 21.7°C was between 2.1 - 4.6°C cooler than other spots.

**C) Plastic (white pot plant) breeding site 2** compared with a spot on a leaf (Sp2) and a spots on concrete ground (Sp3), a spot on rubber mat (Sp4) and spot on plastic container (Sp5). Breeding site temperature 22.9°C was between 5.6 - 7.6°C cooler than other spots.

**Figure: Mid-Hills, Kathmandu (urban) – plastic containers, two *Ae. aegypti* in breeding sites.**

| **A) Image range 20.8 - 30.5°C** | **B) Image range 20.6 - 29.1°C** | **C) Image range 21.7 -31.3°C** |
| --- | --- | --- |
| Sp1 - 23.6 °C breeding site | Sp1 - 21.7 °C breeding site | Sp1 - 22.9 °C breeding site |
| Sp2 - 22.0 °C breeding site | Sp2 - 25.6 °C | Sp2 - 26.2 °C |
| Sp3 - 25.7 °C | Sp3 - 23.8 °C | Sp3 - 29.0 °C |
| Sp4 - 24.0 °C | Sp4 - 23.7 °C | Sp4 - 30.5 °C |
| Sp5 - 23.0 °C | Sp5 - 26.3 °C | Sp5 - 28.5 °C |
| Sp6 - 26.4 °C |  |  |
| Sp7 - 29.7 °C |  |  |

**Case study 2. Metal water container (morning)**

**Location:** Mid**-**Hill Region; Bhaktapur District; Rural

**Dengue vector breeding sites**; Artificial; Metal container, medium size, light colour, >50%

Debris, < 50% sunlight

**Mosquito larvae/pupae**: >300 larvae

**Mosquito species identified**: *Ae. albopictus* and *Cx.* *quinquefasciatus*

**Figure image and temperatures of breeding and non-breeding sites**

**A) Metal (silver) breeding site** – two areas compared including one inside the container with water and identified as a mosquito breeding site (Bx1) and one on the outside of the container (Bx2). Mosquito breeding site (Bx1) with a temperature range of 19.9 - 20.6°C was between 1.5 -3.5°C cooler than the outside of the container.

**Figure: Mid-Hills, Bhaktapur (rural) – metal container and breeding site of *Ae. albopictus* and *Cx.* *quinquefasciatus.***


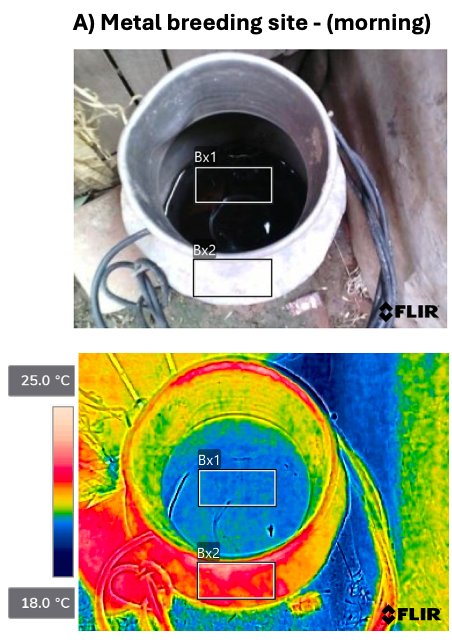


| **A) Image range 18.9 – 24.1°C** |
| --- |
| Bx1 max - 20.6 °C breeding site |
| Bx1 average -20.2°C breeding site |
| Bx1 min - 19.9 °C breeding site |
|  |
| Bx1 max - 24.1 °C |
| Bx1 average - 22.3 °C |
| Bx1 min 21.4 °C |

**Case study 3. Tree water container (morning and midday)**

**Location:** Mountain (lower) Region; Lalitpur District; Rural

**Dengue vector breeding sites**; Natural; tree hole container, small size, light colour, <50% debris, <50% sunlight

**Mosquito larvae/pupae**: 6 pupae

**Mosquito species identified**: *Ae. albopictus*

**Figure image and breeding and temperature site descriptions**

**A) Banana tree** in a household garden, inspected to find a tree branch union containing water.

**B) Tree branch union** **(morning)** identified as a breeding site compared with two spots on adjacent branches without water (Sp2, Sp3). Breeding site temperature 23.1°C was between 0.4 – 0.6°C cooler than branches.

**C) Tree branch union** **(midday)** identified as a breeding site compared with spots on adjacent branches without water (Sp2, Sp3). Breeding site temperature 26.2°C was between 1.2 – 1.6°C cooler than the branches.

The midday breeding site temperature increased 3.1°C from the morning temperature.

**Figure: Mountains, Lalitpur (rural) – natural breeding site of *Ae. albopictus*.**


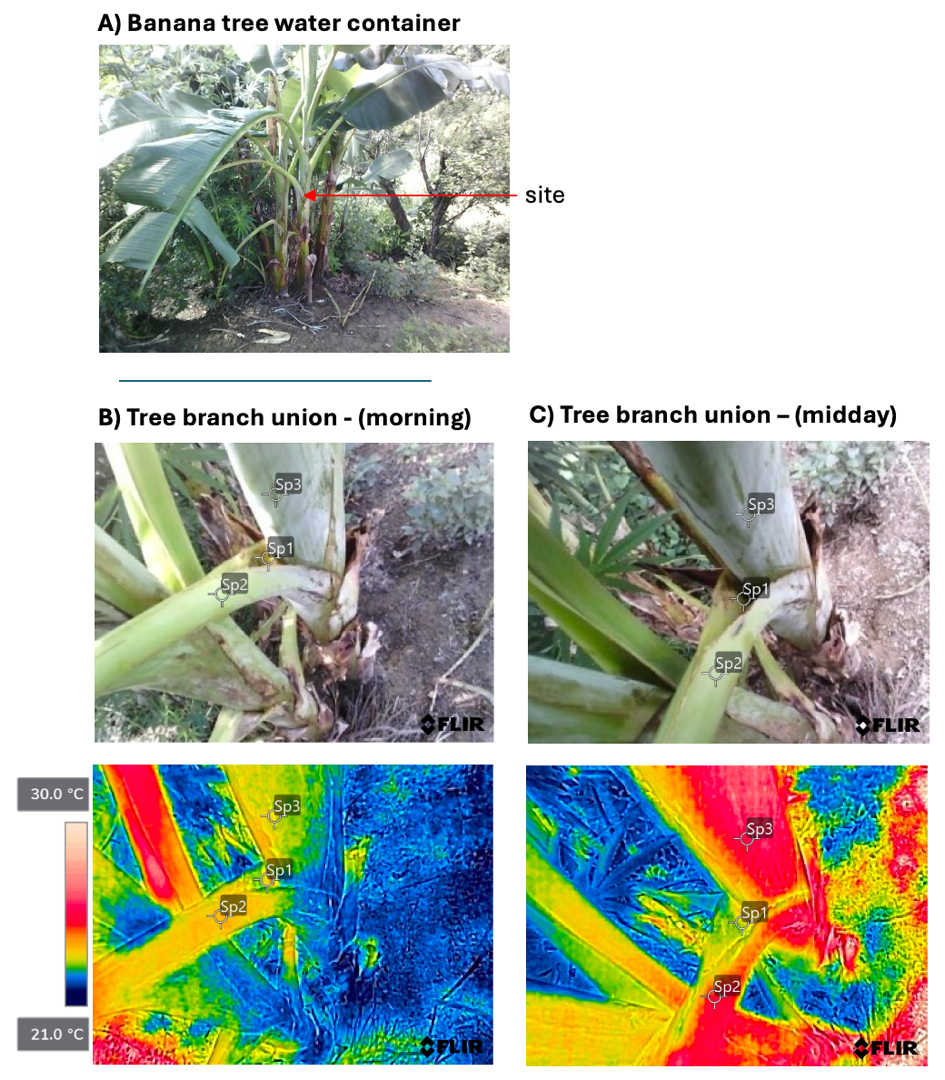


| **B) Image range 21.3 – 27.1°C** | **C) Image range 23.0 – 30.1°C** |
| --- | --- |
| Sp1 - 23.1 °C breeding site | Sp1 - 26.2 °C breeding site |
| Sp2 - 23.7 °C | Sp2 - 27.8 °C |
| Sp3 - 23.5 °C | Sp3 - 27.4 °C |

**Case study 4. Rubber water container (morning and midday)**

**Location:** Mountain Region; Lalitpur District; Rural

**Dengue vector breeding sites**; Artificial; Rubber container breeding site, medium size, dark colour, <50% debris, >50% sunlight

**Mosquito larvae/pupae**: 2 larvae

**Mosquito species identified**: *Ae. lineatopennis*

**Figure image and breeding and temperature site descriptions**

**A) Field and rubber tyres** inspected to find one tyres containing water. One tyre was found to be a breeding site, and two images were examined i) a whole tyre (externally) and ii) close-up of the inner rubber tyre.

**B) Tyre whole** **(morning)** examined with temperature ranging from 18.5 – 22.4°C.

**C) Tyre breeding site (morning)** inner type identified as a breeding site (Sp1) compared with two spots externally without water including the tyre and foliage (Sp2, Sp3). Breeding site temperature of 19.6°C was between 0.3-4.1°C cooler than the external tyre and foliage spots.

**D) Tyre whole** **(midday)** examined with temperature ranging from 25.1 – 57.4°C.

**E) Tyre breeding site (midday)** the inner tyre breeding site (Sp1) compared with two spots externally (Sp2, Sp3) found that the breeding site temperature 28.3°C was 27.7°C cooler than the external tyre spot and 2.4°C warmer than the foliage spot.

The midday breeding site temperate increased 8.7°C from the morning temperature, and the external tyre temperature increased 32.3°C from the morning temperature.

**Figure: Mountain, Lalitpur (rural) – rubber breeding site of *Ae. lineatopennis*.**


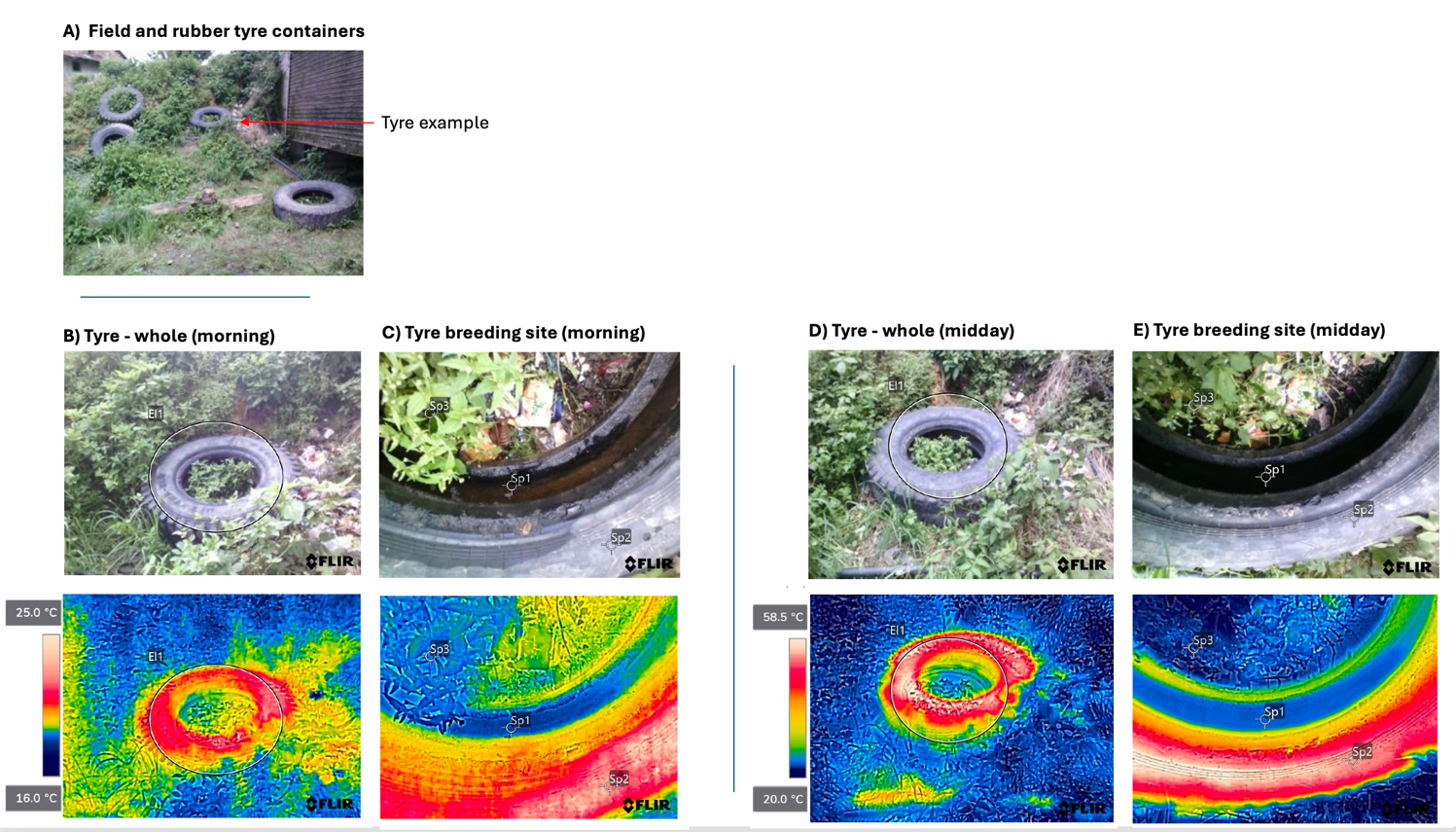


| **B) Image range 16.4– 22.4°C** | **C) Image range 19.0 – 24.3°C** | **D) Image range 21.7– 57.4°C** | **E) Image range 22.4 – 58.5°C** |
| --- | --- | --- | --- |
| El1 max 22.4 °C | Sp1 – 19.6 °C breeding site | El1 max 57.4 °C | Sp1 – 28.3 °C breeding site |
| El1 average 20.1 °C | Sp2 – 23.7 °C | El1 average 41.3 °C | Sp2 – 56.0 °C |
| Ei1 min 18.5 °C | Sp3 – 19.9 °C | Ei1 min 25.1 °C | Sp3 – 25.9 °C |

**Case study 5. Rubber water container (morning and midday)**

**Location:** Terai Region; Chitwan District; Urban

**Dengue vector breeding sites**; Artificial; Rubber container breeding site, medium size, dark colour, <50% debris, >50% sunlight

**Mosquito larvae/pupae**: >300 larvae

**Mosquito species identified**: *Ae. aegypti*

**Figure image and breeding and temperature site descriptions**

**A) Tyre (rubber) breeding site (morning)** – two areas were compared including the inner tyre containing water and identified as a mosquito breeding site (Bx1) and one on the external part of the tyre (Bx2). Mosquito breeding site (Bx1) with a temperature range between 22.1 - 23.4°C was between 0.9 - 1.5°C cooler than the external tyre temperature.

**B) Tyre (rubber) breeding site (midday)** – two areas were compared including the inner tyre containing water and identified as a mosquito breeding site (Bx1) and one on the outside of the tyre (Bx2). Mosquito breeding site (Bx1) with a temperature range between 28.1- 31.1°C was between 4.1 - 14.4°C cooler than the external tyre temperature.

The midday breeding site temperature increased between 6.0 - 7.7°C from the morning temperature and the external tyre temperature increased between 9.1 - 20.9°C from the morning temperature.

**Figure: Terai, Chitwan – rubber breeding site of *Ae. aegypti*.**

**
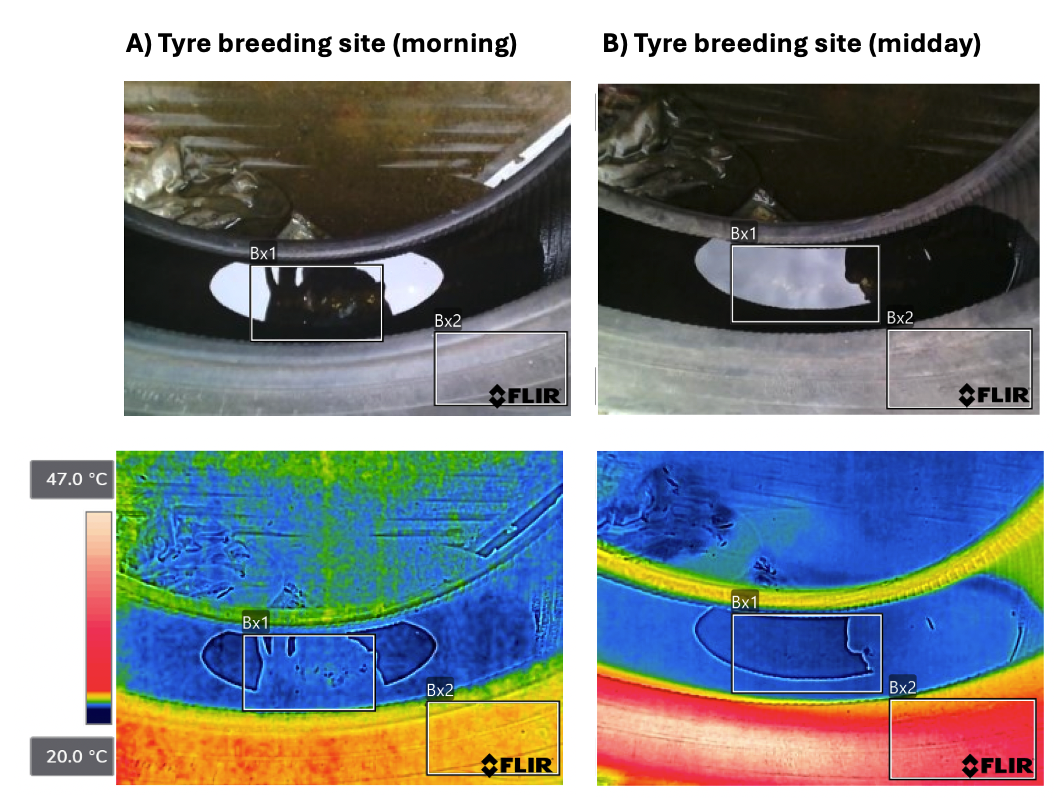
**

| **A) Image range 21.9 – 24.7°C** | **B) Image range 27.0 – 46.5°C** |
| --- | --- |
| Bx1 max - 23.4 °C breeding site | Bx1 max - 31.1 °C breeding site |
| Bx1 average - 22.5 °breeding site | Bx1 average - 28.9 °C breeding site |
| Bx1 min - 22.1 °C breeding site | Bx1 min - 28.1 °C breeding site |
|  |  |
| Bx1 max - 24.3 °C | Bx1 max - 45.2 °C |
| Bx1 average - 23.9 °C | Bx1 average - 43.3 °C |
| Bx1 min -23.0 °C | Bx1 min - 32.1 °C |
